# An Arbitrarily High Order and Asymptotic Preserving Kinetic Scheme in Compressible Fluid Dynamic

**DOI:** 10.1007/s42967-023-00274-w

**Published:** 2023-08-01

**Authors:** Rémi Abgrall, Fatemeh Nassajian Mojarrad

**Affiliations:** Institute of Mathematics, University of Zürich, Winterthurerstrasse 190, CH 8057 Zürich, Switzerland

**Keywords:** Kinetic scheme, Compressible fluid dynamics, High order methods, Explicit schemes, Asymptotic preserving, Defect correction method, 65N99, 76N99

## Abstract

We present a class of arbitrarily high order fully explicit kinetic numerical methods in compressible fluid dynamics, both in time and space, which include the relaxation schemes by Jin and Xin. These methods can use the CFL number larger or equal to unity on regular Cartesian meshes for the multi-dimensional case. These kinetic models depend on a small parameter that can be seen as a “Knudsen” number. The method is asymptotic preserving in this Knudsen number. Also, the computational costs of the method are of the same order of a fully explicit scheme. This work is the extension of Abgrall et al. (2022) [3] to multi-dimensional systems. We have assessed our method on several problems for two-dimensional scalar problems and Euler equations and the scheme has proven to be robust and to achieve the theoretically predicted high order of accuracy on smooth solutions.

## Introduction

In this paper, we consider a system of hyperbolic conservation laws in multiple spatial dimensions

1a $$\begin{aligned} \frac{\partial \textbf{u}}{\partial t}+\sum \limits _{i=1}^d \dfrac{\partial \textbf{A}_i(\textbf{u})}{\partial x_i} =0 \end{aligned}$$

with the initial condition

1b $$\begin{aligned} \textbf{u}(\textbf{x},0)=\textbf{u}_0(\textbf{x}), \end{aligned}$$

where $\textbf{u}\!: {\mathbb {R}}^d\times {\mathbb {R}}_{+}\rightarrow {\mathbb {R}}^K$ and the flux functions $\textbf{A}_d$ are locally Lipschitz continuous on ${\mathbb {R}}^K$ with values in ${\mathbb {R}}^K$. We approximate the solution $\textbf{u}$ by considering a special class of discrete kinetic systems [19, 22].

Consider a solution $\textbf{f}\!:{\mathbb {R}}^d\times {\mathbb {R}}_{+}\rightarrow {\mathbb {R}}^{L}$ to the Cauchy problem for the following sequence of semilinear systems:

2a $$\begin{aligned} \frac{\partial \textbf{f}}{\partial t}+\sum \limits _{i=1}^d\Lambda _i \frac{\partial \textbf{f}}{\partial x_i}=\frac{{\mathbb {M}}(\textbf{u}^{\varepsilon })-\textbf{f}}{\varepsilon } \end{aligned}$$

with the initial condition

2b $$\begin{aligned} \textbf{f}(\textbf{x},0)=\textbf{f}_0(\textbf{x}). \end{aligned}$$

Here $\Lambda _i$ are real diagonal $L\times L$ matrices, $\varepsilon$ is a positive number, ${\mathbb {M}}\!:{\mathbb {R}}^K\rightarrow {\mathbb {R}}^L$ is a Lipschitz continuous function, and the function $\textbf{u}^{\varepsilon }$ is defined by

$$\begin{aligned} \textbf{u}^{\varepsilon }=\sum \limits _{i=1}^N \textbf{f}_i ={\mathbb {P}}\textbf{f},\end{aligned}$$

where ${\mathbb {P}}$ is a real constant coefficients $K \times L$ matrix. To connect problem (2a)–(2b) with problem (1a)–(1b), we assume that ${\mathbb {M}}$ is a Maxwellian function for (1a), i.e.,

3 $$\begin{aligned} {\left\{ \begin{array}{ll} {\mathbb {P}}{\mathbb {M}}(\textbf{u})=\textbf{u},\\ {\mathbb {P}}\Lambda _i {\mathbb {M}}(\textbf{u})=\textbf{A}_i(\textbf{u}),\quad i=1, \cdots , d. \end{array}\right. } \end{aligned}$$

Clearly, if $\textbf{f}$ converges in some strong topology to a limit $\textbf{g}$ and if ${\mathbb {P}}\textbf{f}_0$ converges to $\textbf{u}_0$, then ${\mathbb {P}}\textbf{g}$ is a solution of problem (1a)–(1b). Actually, the system (2a) is only a BGK approximation for (1a), see e.g., [6, 10] and the references therein. A general stability theory was developed in [9], and will implicitly be used throughout this paper, in particular to guarantee that the continuous problem (2), equipped with (3) is well posed.

The method of [4, 19], where the first numerical schemes based on (2) are described, is based on splitting techniques. As a result, the order in time is restricted to 2 and can only be improved by nontrivial manipulations [12]. There exist already some ways for higher than second order. For example, one approach is to use relaxed upwind schemes which are proposed to running up to CFL 1 and up to third order in time and space for the finite volume scheme [23]. A class of high-order weighted essentially nonoscillatory (WENO) reconstructions based on relaxation approximation of hyperbolic systems of conservation laws was presented in [5]. In [20] a splitting approach was adopted with a regular CFL stability condition for the overall finite volume scheme. In [11] a discontinuous Galerkin method for solving general hyperbolic systems of conservation laws was constructed which is CFL independent and can be of arbitrary order in time and space.

Designing algorithms that are uniformly stable and acccurate in $\varepsilon$ when $\varepsilon \rightarrow 0$ has been an active area of research in recent years. Such schemes are called asymptotic preserving in the sense of Jin [18]. Several asymptotic-preserving methods based on IMEX techniques have been recently proposed. In [7, 8] IMEX Runge-Kutta methods were presented for general hyperbolic systems with relaxation. Specific methods for the Boltzmann equation in the hyperbolic and diffusive regimes with a computational cost that is independent of $\varepsilon$ were proposed in [13, 15].

Many of these methods have inherent limitations with respect to the order that can be achieved with the time discretization, for example, due to the time splitting. In [3] an arbitrarily high order class of kinetic numerical methods that can run at least at CFL 1 in one dimension was constructed. This work is an extension of [3] to the multi-dimensional case.

We are interested in a computationally *explicit* scheme that solves (2) with uniform accuracy of order $r > 0$ for all $\varepsilon > 0$ and with a CFL condition, based on the matrices $\Lambda _i,~i=1, \cdots , d$, that is larger than one for a given system (1). We consider the two-dimensional case. The idea is to start from (2a), we describe first the discretisation of $\Lambda _x \frac{\partial \textbf{f}}{\partial x}$ and $\Lambda _y \frac{\partial \textbf{f}}{\partial y}$. The second step is to discretise in time. We take into account the source term. The resulting scheme is fully implicit. The next step is to show that, thanks to the operator ${\mathbb {P}}$, and using a particular time discretisation, we can make it computationally explicit, and high order accurate. It is independent of $\varepsilon$.

The paper is organised as follows. In Sect. 2, we describe the time-stepping algorithm. In Sect. 3, we describe the space discretisation. In Sect. 4, We study the stability of the discretisation of the homogeneous problem. In Sect. 5, we illustrate the robustness and accuracy of the proposed method by means of numerical tests. Finally, Sect. 6 provides some conclusions and future perspectives.

## Time Discretisation

Here, we consider the two-dimensional case, i.e.,

4 $$\begin{aligned} \frac{\partial \textbf{f}}{\partial t}+\Lambda _x \frac{\partial \textbf{f}}{\partial x}+\Lambda _y \frac{\partial \textbf{f}}{\partial y}=\frac{{\mathbb {M}}({\mathbb {P}}\textbf{f})-\textbf{f}}{\varepsilon }. \end{aligned}$$

Knowing the solution $\textbf{f}^{n}$ at time $t_n$, we are looking for the solution at time $t_{n+1}$. First we discretise (2a) in space, we get

5 $$\begin{aligned} \frac{\partial \textbf{f}}{\partial t}+\frac{1}{\Delta x}\Lambda _x \delta ^x\textbf{f}+\frac{1}{\Delta y}\Lambda _y \delta ^y\textbf{f}=\frac{{\mathbb {M}}({\mathbb {P}}\textbf{f})-\textbf{f}}{\varepsilon }, \end{aligned}$$

and notice that

6 $$\begin{aligned} \frac{\partial {\mathbb {P}}\textbf{f}}{\partial t}+\frac{1}{\Delta x}{\mathbb {P}}(\Lambda _x \delta ^x\textbf{f})+\frac{1}{\Delta y}{\mathbb {P}}(\Lambda _y \delta ^y\textbf{f})=0. \end{aligned}$$

Since we want to have a running CFL number of at least one, we use an IMEX defect correction method. Using this, we follow [3] where a defect correction technique can be used and it is made explicit because the nonlinear term ${\mathbb {M}}({\mathbb {P}}\textbf{f})$ is explicit.

### An Explicit High Order Timestepping Approach

The next step is to discretise in time, we subdivide the interval $[t_n,t_{n+1}]$ into sub-intervals obtained from the partition

$$\begin{aligned} t_n=t_{(0)}<t_{(1)}<\cdots t_{(p)}<\cdots <t_{(M)}=t_{n+1} \end{aligned}$$

with $t_{(p)}=t_n+\beta _p\Delta t$. We approximate the integral over time using the quadrature formula

$$\begin{aligned} \int _{t_n}^{t_n+\beta _p\Delta t} \phi (s) \text ds\approx \Delta t\sum _{q=0}^M w_{pq}\phi (t_n+\beta _q\Delta t), \end{aligned}$$

where $w_{pq}$ are the weights. In order to obtain consistent quadrature formula of order $q+1$, we should have

$$\begin{aligned} w_{pq}=\int _0 ^{\beta _p} l_q(s) \text ds,\quad \sum _{q=0} ^M w_{pq}=\beta _p, \end{aligned}$$

where $\{l_q\}_{q=0} ^M$ is the Lagrangian basis polynomial of the *q*-th node.

Let $\textbf{x}_l=(x_i,y_j)$ be a fixed grid point, $\textbf{f}_l ^{n,p}\approx \textbf{f}(\textbf{x}_l,t_n+\beta _p \Delta t)$ and $\textbf{f}_l ^{n,0}=\textbf{f}_l ^{n}$. We introduce the corrections $r=0, \cdots , R$ for each subinterval $[t_p,t_{p+1}]$ and denote the solution at the *r*-th correction and the time $t_p$ by $\textbf{f}^{n,p,r}$. The notation $\textbf{F}$ is the collection of all the approximations for the sub-steps i.e., the vector $\textbf{F}=(\textbf{f}^{n,1},\cdots ,\textbf{f}^{n,M})^\text{T}$. The notation $\textbf{F}^{(r)}$ represents the vector $\textbf{F}^{(r)}=(\textbf{f}^{n,1,r},\cdots ,\textbf{f}^{n,M,r})^\text{T}$, i.e., the vector of all the approximations for the sub-steps at the *r*-th correction. Now we use a defect correction method and proceed within the time interval $[t_n, t_{n+1}]$ as follows.

(i) For $$r=0$$, set $$\textbf{F}^{(0)}=(\textbf{f}^{n},\cdots ,\textbf{f}^{n})^\text{T}$$.
(ii) For each correction $r \geqslant 0$, define $\textbf{F}^{(r+1)}$ by
  7 $$\begin{aligned} L_1(\textbf{F}^{(r+1)})=L_1(\textbf{F}^{(r)})-L_2(\textbf{F}^{(r)}) \end{aligned}.$$
(iii) Set $$\textbf{F}^{n+1}=\textbf{F}^{(M)}$$.

Formulation (7) relies on a Lemma which has been proven in [1].

In the following, we introduce the differential operators $L_1$ and $L_2$. We first define the high order differential operator $L_2$. By integrating (5) on [0, *t*], we have

8 $$\begin{aligned}{} & {} \textbf{f}(\textbf{x},t)-\textbf{f}(\textbf{x},0)+\frac{1}{\Delta x} \int _0 ^t \Lambda _x \delta ^x\textbf{f}(\textbf{x},s)  \text ds\nonumber \\{} & {} +\frac{1}{\Delta y} \int _0 ^t \Lambda _y \delta ^y\textbf{f}(\textbf{x},s) \text ds=\frac{1}{\varepsilon }\int _0 ^t \Big ({\mathbb {M}}\big ({\mathbb {P}}\textbf{f}(\textbf{x},s)\big )-\textbf{f}(\textbf{x},s)\Big ) \text ds. \end{aligned}$$

Hence, we get the following approximation for (4):

9 $$\begin{aligned}{} & {} \textbf{f}_l ^{n,q}-\textbf{f}_l ^{n,0}+\frac{\Delta t}{\Delta x}\left (\sum _{k=0} ^M w_{qk}\Lambda _x\delta _l ^x\textbf{f}^{n,k} \right )+\frac{\Delta t}{\Delta y}\left (\sum _{k=0} ^M w_{qk}\Lambda _y\delta _l ^y\textbf{f}^{n,k} \right )\nonumber \\{} & {} -\frac{\Delta t}{\varepsilon }\sum _{k=0} ^M w_{qk}\left ({\mathbb {M}}({\mathbb {P}} \textbf{f}_l ^{n,k})-\textbf{f}_l ^{n,k}\right )=0 \end{aligned}$$

for $q=1, \cdots ,M$. For any *l*, define $[L_2(\textbf{F}^{(r)})]_l$ as

$$\begin{aligned}[L_2(\textbf{F}^{(r)})]_{l}=\,&\textbf{F}_l^{(r)}-\textbf{F}_l ^{(0)}+\frac{\Delta t}{\Delta x}\Lambda _xW\delta _l ^x \textbf{F}^{(r)}\\&+\frac{\Delta t}{\Delta x}\Lambda _x \textbf{w}_0\otimes \delta _l ^x \textbf{f}^{n,0}+\frac{\Delta t}{\Delta y}\Lambda _yW\delta _l ^y \textbf{F}^{(r)}+\frac{\Delta t}{\Delta y}\Lambda _y \textbf{w}_0\otimes \delta _l ^y \textbf{f}^{n,0}\\&-\frac{\Delta t}{\varepsilon }W\big ( {\mathbb {M}}({\mathbb {P}}\textbf{F}_l^{(r)})-\textbf{F}_l^{(r)}\big )-\frac{\Delta t}{\varepsilon }\textbf{w}_0\otimes \big ( {\mathbb {M}}({\mathbb {P}}\textbf{f}_l ^{n,0})-\textbf{f}_l ^{n,0}\big ), \end{aligned}$$

where

$$\begin{aligned} W = \begin{pmatrix} w_{11} &{} \cdots &{} w_{1M} \\ \vdots &{}  &{} \vdots \\ w_{M1} &{} \cdots &{} w_{MM} \\ \end{pmatrix},\quad \textbf{w}_0=\begin{pmatrix} w_{10}\\ \vdots \\ w_{M0} \end{pmatrix}, \end{aligned}$$

and

$$\begin{aligned} {\mathbb {M}}({\mathbb {P}}\textbf{F}_l^{(r)})=\big ({\mathbb {M}}({\mathbb {P}}\textbf{f}_l^{n,1,r}),\cdots ,{\mathbb {M}}({\mathbb {P}}\textbf{f}_l ^{n,M,r})\big )^{\text{T}}. \end{aligned}$$

The resulting scheme derived by the $L_2$ operator is implicit, and it is very difficult to solve. Now we describe the low order differential operator $L_1$. We use the forward Euler method on each sub-time step

10 $$\begin{aligned} \textbf{f}_l ^{n,q}-\textbf{f}_l ^{n,0}+\beta _q\frac{\Delta t}{\Delta x}\Lambda _x\delta _l ^x\textbf{f}^{n,0} +\beta _q\frac{\Delta t}{\Delta y}\Lambda _y\delta _l ^y\textbf{f}^{n,0} -\frac{\Delta t}{\varepsilon }\sum _{k=0} ^M w_{qk}\big ({\mathbb {M}}({\mathbb {P}} \textbf{f}_l ^{n,k})-\textbf{f}_l ^{n,k}\big )=0 \end{aligned}$$

for $q=1,\cdots ,M$. The low order differential operator $[L_1(\textbf{F}^{(r)})]_l$ reads

$$\begin{aligned}{}[L_1(\textbf{F}^{(r)})]_l=\,&\textbf{F}_l^{(r)}-\textbf{F}_l ^{(0)}+\frac{\Delta t}{\Delta x}B\Lambda _x\delta _l ^x \textbf{F}^{(0)}+\frac{\Delta t}{\Delta y}B\Lambda _y\delta _l ^y \textbf{F}^{(0)}\\&-\frac{\Delta t}{\varepsilon }W\big ( {\mathbb {M}}({\mathbb {P}}\textbf{F}^{(r)}_l)-\textbf{F}_l^{(r)}\big )-\frac{\Delta t}{\varepsilon }\textbf{w}_0\otimes \big ( {\mathbb {M}}({\mathbb {P}}\textbf{f}_l ^{n,0})-\textbf{f}_l^{n,0}\big ), \end{aligned}$$

where $B=\text {diag}(\beta _1, \cdots , \beta _M)$. This is still an implicit approximation in time, but the convection part is now explicit. We also note we have kept the same form of the source term approximation in both cases, for reasons that will be explained bellow.

Now we can write (7) as a multi-step method where each step writes as

11 $$\begin{aligned} \begin{aligned}&\textbf{F}_l^{(r+1)}-\textbf{F}_l ^{(0)}+\frac{\Delta t}{\Delta x}B\Lambda _x\delta _l ^x \textbf{F}^{(0)}+\frac{\Delta t}{\Delta y}B\Lambda _y\delta _l ^y \textbf{F}^{(0)} -\frac{\Delta t}{\varepsilon }W\big ( {\mathbb {M}}({\mathbb {P}}\textbf{F}^{(r+1)}_l)-\textbf{F}_l^{(r+1)}\big ) \\&-\frac{\Delta t}{\varepsilon }\textbf{w}_0\otimes \big ( {\mathbb {M}}({\mathbb {P}}\textbf{f}_l ^{n,0})-\textbf{f}_l^{n,0}\big ) =\frac{\Delta t}{\Delta x}\Lambda _xW(\delta _l ^x \textbf{F}^{(0)}-\delta _l ^x \textbf{F}^{(r)})+\frac{\Delta t}{\Delta y}\Lambda _yW(\delta _l ^y \textbf{F}^{(0)}-\delta _l ^y \textbf{F}^{(r)}) \end{aligned} \end{aligned}$$

for any *l*. By applying ${\mathbb {P}}$ to this equation, we will obtain the following equation for calculating ${\mathbb {P}}\textbf{F}_l^{(r+1)}$:

12 $$\begin{aligned}{}  {} {\mathbb {P}}\textbf{F}_l^{(r+1)}=\,&{\mathbb {P}}\textbf{F}_l^{(0)}-\frac{\Delta t}{\Delta x}{\mathbb {P}}\Lambda _xW\delta _l ^x \textbf{F}^{(r)}-\frac{\Delta t}{\Delta x}\textbf{w}_0\otimes {\mathbb {P}} \Lambda _x\delta _l ^x \textbf{f}^{n,0}\nonumber \\{} & {} -\frac{\Delta t}{\Delta y}{\mathbb {P}}\Lambda _yW\delta _l ^y \textbf{F}^{(r)}-\frac{\Delta t}{\Delta y}\textbf{w}_0\otimes {\mathbb {P}} \Lambda _y\delta _l ^y \textbf{f}^{n,0}, \end{aligned}$$

and we substitute ${\mathbb {P}}\textbf{F}_l^{(r+1)}$ into the Maxwellian in (11). Alternatively, one can rewrite (11) as follows:

13 $$\begin{aligned} \begin{aligned}\left(\text {Id}_{M\times M}+\frac{\Delta t}{\varepsilon }W\right)\textbf{F}_l^{(r+1)}=\,&\frac{\Delta t}{\varepsilon }W{\mathbb {M}}({\mathbb {P}}\textbf{F}_l^{(r+1)}) \\&+\textbf{F}_l^{(0)}-\frac{\Delta t}{\Delta x}\Lambda _xW\delta _l ^x \textbf{F}^{(r)} -\frac{\Delta t}{\Delta x}\textbf{w}_0\otimes \Lambda _x\delta _l ^x \textbf{f}^{n,0}\\&-\frac{\Delta t}{\Delta y}\Lambda _yW\delta _l ^y \textbf{F}^{(r)} -\frac{\Delta t}{\Delta y}\textbf{w}_0\otimes \Lambda _y\delta _l ^y \textbf{f}^{n,0}+\frac{\Delta t}{\varepsilon }\textbf{w}_0\otimes \big ( {\mathbb {M}}({\mathbb {P}}\textbf{f}_l ^{n,0})-\textbf{f}_l^{n,0}\big ). \end{aligned} \end{aligned}$$

If $\text {Id}_{M\times M}+{\Delta t}W/{\varepsilon }$ is invertible, the defect correction computes the solution at time $t_{n+1}$ using *M* steps of the form

14a $$\begin{aligned} \begin{aligned}\textbf{F}_l^{(r+1)}=&\left(\text {Id}_{M\times M}+\frac{\Delta t}{\varepsilon }W\right)^{-1}\bigg ( \frac{\Delta t}{\varepsilon }W{\mathbb {M}}({\mathbb {P}}\textbf{F}_l^{(r+1)})\\&+\textbf{F}_l^{(0)}-\frac{\Delta t}{\Delta x}\Lambda _xW\delta _l ^x \textbf{F}^{(r)} -\frac{\Delta t}{\Delta x}\textbf{w}_0\otimes \Lambda _x\delta _l ^x \textbf{f}^{n,0}\\&-\frac{\Delta t}{\Delta y}\Lambda _yW\delta _l ^y \textbf{F}^{(r)} -\frac{\Delta t}{\Delta y}\textbf{w}_0\otimes \Lambda _y\delta _l ^y \textbf{f}^{n,0}+\frac{\Delta t}{\varepsilon }\textbf{w}_0\otimes \big ( {\mathbb {M}}({\mathbb {P}}\textbf{f}_l ^{n,0})-\textbf{f}_l^{n,0}\big ) \bigg ). \end{aligned} \end{aligned}$$

For computing ${\mathbb {P}}\textbf{F}_l ^{(r+1)}$, for any *l*, we rewrite (12) as (for simplicity, we drop the superscript *n*)

14b $$\begin{aligned} {\mathbb {P}}\textbf{f}_l ^{q,r+1}-{\mathbb {P}}\textbf{f}_l ^{0}+\frac{\Delta t}{\Delta x}\sum _{k=0} ^M w_{qk}{\mathbb {P}}\Lambda _x\delta _l ^x\textbf{f}^{k,r} +\frac{\Delta t}{\Delta y}\sum _{k=0} ^M w_{qk}{\mathbb {P}}\Lambda _y\delta _l ^y\textbf{f}^{k,r}=0 \end{aligned}$$

for $$q=1, \cdots , M$$.

We write the increment $\delta _l \textbf{f}^{k}$ as a sum of residuals as follows:

15a $$\begin{aligned} \begin{aligned} \delta _l \textbf{f}^k&=\Delta y\Lambda _x({\hat{\textbf{f}}}_{i+\frac{1}{2},j}^k-{\hat{\textbf{f}}}_{i-\frac{1}{2},j}^k) +\Delta x\Lambda _y({\hat{\textbf{f}}}_{i,j+\frac{1}{2}}^k-{\hat{\textbf{f}}}_{i,j-\frac{1}{2}}^k)\\&=\Phi _{l,(i,j)} ^{[i,i+1]\times [j,j+1],k}+ \Phi _{l,(i,j)} ^{[i-1,i]\times [j,j+1],k}+ \Phi _{l,(i,j)} ^{[i,i+1]\times [j-1,j],k}+ \Phi _{l,(i,j)} ^{[i-1,i]\times [j-1,j],k} \end{aligned} \end{aligned}$$

with

15b $$\begin{aligned} \left\{ \begin{aligned} &\Phi _{l,(i,j)} ^{[i,i+1]\times [j,j+1],k}=\frac{1}{2}\bigg (\Lambda _x \left ( \hat{\textbf{f}}_{i+1/2,j}-\textbf{f}_{ij}\right)\Delta y+ \Lambda _y\big (\hat{\textbf{f}}_{i,j+1/2}-\textbf{f}_{ij}\big )\Delta y\bigg ), \\ &\Phi _{l,(i,j)} ^{[i-1,i]\times [j,j+1],k}=\frac{1}{2}\bigg (\Lambda _x \big (\hat{\textbf{f}}_{i+1/2,j}-\textbf{f}_{ij}\big )\Delta y+ \Lambda _y\left(\textbf{f}_{ij}-\hat{\textbf{f}}_{i,j-1/2}\right)\Delta x\bigg ), \\ &\Phi _{l,(i,j)} ^{[i-1,i]\times [j-1,j],k}= \frac{1}{2}\bigg ( \Lambda _x\big (\textbf{f}_{ij}-\hat{\textbf{f}}_{i-1/2,j}\big )\Delta y+\Lambda _y\big ( \textbf{f}_{ij}-\hat{\textbf{f}}_{ij-1/2}\big )\Delta x \bigg ), \\ &\Phi _{l,(i,j)} ^{[i-1,i]\times [j-1,j],k}=\frac{1}{2}\bigg ( \Lambda _x\big (\textbf{f}_{ij}-\hat{\textbf{f}}_{i-1/2,j}\big )\Delta y+\Lambda _y\big ( \hat{\textbf{f}}_{i,j+1/2}-\textbf{f}_{ij}\big ) \Delta x\bigg ). \\ \end{aligned} \right.\end{aligned}$$

**Fig. 1:**
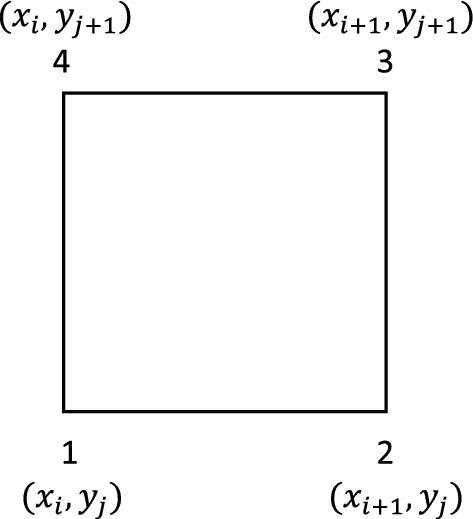
Sample quad element

We see that

16 $$\begin{aligned} \begin{aligned} &\Phi _{l,(i,j)} ^{[i,i+1]\times [j,j+1],k}+\Phi _{l,(i+1,j)} ^{[i,i+1]\times [j,j+1],k}+\Phi _{l,(i+1,j+1)} ^{[i,i+1]\times [j,j+1],k}+\Phi _{l,(i,j+1)} ^{[i,i+1]\times [j,j+1],k}\\=& \frac{\Delta x}{2}\Lambda _x\big ( \textbf{f}_{i+1,j}+\textbf{f}_{i+1,j+1}\big )-\frac{\Delta x}{2}\Lambda _x\big (\textbf{f}_{ij}+\textbf{f}_{i,j+1}\big ) \\&+\frac{\Delta y}{2}\Lambda _y\big ( \textbf{f}_{i+1,j}+\textbf{f}_{i+1,j+1}\big )-\frac{\Delta y}{2}\Lambda _y\big ( \textbf{f}_{ij}+\textbf{f}_{i,j+1}\big ),\end{aligned} \end{aligned}$$

i.e., the sum of the residuals assigned to each of the four vertices of the quad $[x_{i},x_{i+1}]\times [y_j,y_{j+1}]$ is equal to an approximation of the flux across the boundary of this quad; see Fig. 1. This guarantees the local conservation; see [2]. The reason of writing the space update as the sum of residual will become clear in Sect. 3.2.

Now we explain the residuals for an example where the velocities are orthogonal. This case was suggested in [4]. In order to construct the system (2a) one must find ${\mathbb {P}}$, ${\mathbb {M}}$, and $\Lambda _j ~(j=1,\cdots ,d)$ such that the consistency relations (3) are satisfied. We take $L=N \times K$, ${\mathbb {P}}=(\text {Id}_{K\times K}, \cdots ,\text {Id}_{K\times K})$ with *N* blocks $\text {Id}_{K\times K}$, the identity matrix in ${\mathbb {R}}^K$. Each matrix $\Lambda _j~ (j=1,\cdots ,d)$ is constituted of *N* diagonal blocks of size $K\times K$, i.e.,

$$\begin{aligned} \Lambda _j=\text {diag}(C_1^{(j)},\cdots ,C_N^{(j)}),\quad C_i^{(j)}=\lambda _i^{(j)}\text {Id}_{K\times K},\quad \lambda _i^{(j)}\in {\mathbb {R}}, \end{aligned}$$

where $j=1,\cdots ,N$. Then, (2a) can be rewritten as

17 $$\begin{aligned} \frac{\partial \textbf{f}_j}{\partial t}+\sum \limits _{i=1}^d\lambda _j^{(i)} \frac{\partial \textbf{f}_j}{\partial x_i}=\frac{{\mathbb {M}}_j(\textbf{u}^{\varepsilon })-\textbf{f}_j}{\varepsilon },\, j=1,\cdots ,N \end{aligned}$$

in which $\textbf{u}^{\varepsilon }=\sum \limits _{j=1}^N \textbf{f}_n$. This example works with any number of blocks provided that $N\geqslant d+1$. We take the velocities such that

$$\begin{aligned} \Lambda _j=\text {diag}(\lambda _{ij}\text {Id} _{K\times K})_{1 \leqslant i \leqslant N},\quad  \sum _{i=1} ^{N} \lambda _{ij}=0,~ j=1,\cdots , d, \end{aligned}$$

and

$$\begin{aligned} \sum _{i=1} ^{N} \lambda _{il}\lambda _{ij}=0,~ j,l=1,\cdots, d,~ j\ne l. \end{aligned}$$

Therefore, the Maxwellian function is given by

$$\begin{aligned} {\mathbb {M}}_{i}(\textbf{u})=\frac{\textbf{u}}{N}+\sum _{j=1} ^d \frac{\textbf{A}_j(\textbf{u})}{a_j ^2} \lambda _{ij},~i=1,\cdots ,N, \end{aligned}$$

where $a_j ^2=\sum _{i=1} ^{N} \lambda _{ij}^2$. We fix $J,N'\geqslant 1$, $\lambda >0$ and set

$$\begin{aligned} {\left\{ \begin{array}{ll} \lambda _j=\frac{n}{J}\lambda, &{}1 \leqslant j\leqslant J,~J\geqslant 1,\\ \Lambda _x=\text {diag}\Big (\lambda _n\text {diag}\Big ( \cos \left(\frac{i\uppi }{2N^{'}}\right)\text { I} \Big )_{1\leqslant i\leqslant 4N^{'}}\Big ),\, &{}N^{'}\geqslant 1, \\ \Lambda _y=\text {diag}\Big (\lambda _n\text {diag}\Big ( \sin \left(\frac{i\uppi }{2N^{'}}\right)\text { I} \Big )_{1\leqslant i\leqslant 4N^{'}}\Big ). \end{array}\right. } \end{aligned}$$

Here we have $N=4N'J$. We define the Maxwellian functions by

$$\begin{aligned} {\mathbb {M}}_{j}(\textbf{u})=\frac{1}{N}\left( \textbf{u}+\frac{12iJ}{\lambda (J+1)(2J+1)}\left(\textbf{A}_1\cos \frac{j\uppi }{2N^{'}}+\textbf{A}_2\sin \frac{j\uppi }{2N^{'}}\right) \right ), ~1\leqslant j\leqslant 4N^{'}, 1\leqslant i\leqslant J. \end{aligned}$$

Now, we consider the special case where $J=N'=1$ and $N=4$. Set

$$\begin{aligned} {\left\{ \begin{array}{ll} \Lambda _x=\text {diag}\Big (\lambda \text { diag}\Big ( \cos \left(\frac{i\uppi }{2}\right)\text { I} \Big )_{1\leqslant i\leqslant 4}\Big ), \\ \Lambda _y=\text {diag}\Big (\lambda \text { diag}\Big ( \sin \left(\frac{i\uppi }{2}\right)\text { I} \Big )_{1\leqslant i\leqslant 4}\Big ). \end{array}\right. } \end{aligned}$$

Therefore, the Maxwellian functions are

$$\begin{aligned} {\mathbb {M}}_{i}(\textbf{u})=\frac{1}{4}\Big ( \textbf{u}+\frac{2}{\lambda }\left(\textbf{A}_1\cos \frac{i\uppi }{2}+\textbf{A}_2\sin \frac{i\uppi }{2}\right) \Big ) \end{aligned}$$

for $$1\leqslant i \leqslant 4$$.

### Error Estimate

The natural question is: how many iterations should we do in the method (14) to recover the time and space accuracy?

Again, we will consider the two-dimensional version of (1a). Let us consider $\varphi{:} {\mathbb {R}}^2\rightarrow {\mathbb {R}}^K$, which is continuously differentiable on ${\mathbb {R}}^2$ with values in ${\mathbb {R}}^K$ and has compact support. We will consider the discrete version of $L^2$ and $H^1$ norms of $\varphi$ as follows:

$$\begin{aligned}{} & {} \Vert \varphi \Vert _{L^2}^2 = \sum \limits _{i,j\in {\mathbb {Z}}}\Delta x\Delta y \Vert \varphi _{i,j} \Vert ^2,\quad \Vert \varphi \Vert _{H^1}^2  {} =\Vert \varphi \Vert _{L^2}^2+\sum \limits _{i,j\in {\mathbb {Z}}}\Delta x\Delta y \Big (\Big \Vert \dfrac{\varphi _{i+1,j}-\varphi _{ij}}{\Delta x} \Big \Vert ^2+\Big \Vert \dfrac{\varphi _{i,j+1}-\varphi _{i,j}}{\Delta y} \Big \Vert ^2\Big ). \end{aligned}$$

In the following, we will set

$$\begin{aligned} D_{i+1/2,j}\varphi =\dfrac{\varphi _{i+1,j}-\varphi _{ij}}{\Delta x}, D_{i,j+1/2}\varphi =\dfrac{\varphi _{i,j+1}-\varphi _{i,j}}{\Delta y},\text { and } D\varphi _{ij}=(D_{i+1/2,j}\varphi ,D_{i,j+1/2}\varphi ). \end{aligned}$$

We have a Poincaré inequality, on any compact.

One can consider the discrete equivalent of $L^2_\text{loc}$ and $H^{-1}_\text{loc}$ in an interval $I=[a,b]\times [c,d]$ as follows:

$$\begin{aligned} \Vert \textbf{F}\Vert _{2,I}=\sup \limits _{\varphi \in C_0^1(I)^K} \frac{\sum \limits _{i,j\in {\mathbb {Z}}}\Delta x\Delta y \langle \varphi _{i,j},\textbf{f}_{i,j} \rangle }{\Vert \varphi \Vert _{L^2}},\quad \Vert \textbf{F}\Vert _{-1,I}=\sup \limits _{\varphi \in C_0^1(I)^K} \frac{\sum \limits _{i,j\in {\mathbb {Z}}}\Delta x\Delta y \langle \varphi _{i,j},\textbf{f}_{i,j} \rangle }{\Vert \varphi \Vert _{H^1}}. \end{aligned}$$

We have a first result on the $H^{-1}_\text{loc}$ estimate of $L_2(\textbf{F})-L_1(\textbf{F})$.

#### Lemma 1

If ${\widehat{\textbf{F}}}_{i+\frac{1}{2},j}=\sum \limits _{l=-r}^s\alpha _l \textbf{F}_{i+l,j}$ and ${\widehat{\textbf{F}}}_{i,j+\frac{1}{2}}=\sum \limits _{l=-r'}^{s'}\alpha '_l \textbf{F}_{i,j+l}$, we have

$$\begin{aligned} \Vert L_2(\textbf{F})-L_1(\textbf{F})\Vert _{-1,I}\leqslant \gamma \Delta t\Vert \textbf{F}\Vert _{2,I}, \end{aligned}$$

where $$\gamma =\max \big\{\max \limits _{-r\leqslant l\leqslant s} |\alpha _l|,\max \limits _{-r\leqslant l\leqslant s'}|\alpha '_l|\big\}\times \max \big\{\max \limits _m|\lambda _m^x|,\max \limits _n|\lambda _n^y| \big\}$$.

#### Proof

$$\begin{aligned}&\Big |\sum \limits _{i,j}\Delta x\Delta y \Big\langle \varphi _{i,j},[L_2(\textbf{F})]_{i,j}-[L_1(\textbf{F})]_{i,j} \Big\rangle \Big |^2\\ {}=&\Big |\sum \limits _{i,j} \Delta t\Delta y \Big\langle \varphi _{i,j},\Lambda _xW\left({\widehat{\textbf{F}}}_{i+\frac{1}{2},j}-{\widehat{\textbf{F}}}_{i-\frac{1}{2},j}\right) \Big\rangle +\sum \limits _{i,j} \Delta t\Delta x \Big\langle \varphi _{i,j},\Lambda _yW\left({\widehat{\textbf{F}}}_{i,j+\frac{1}{2}}-{\widehat{\textbf{F}}}_{i,j-\frac{1}{2}}\right) \Big\rangle \Big |^2\\=&\Big |\sum \limits _{i,j} \Delta t\Delta x\Delta y \Big\langle D_{i+\frac{1}{2},j}\varphi ,\Lambda _xW{\widehat{\textbf{F}}}_{i+\frac{1}{2},j} \Big\rangle +\sum \limits _{i,j} \Delta t\Delta x\Delta y \Big\langle D_{i,j+\frac{1}{2}}\varphi ,\Lambda _yW{\widehat{\textbf{F}}}_{i,j+\frac{1}{2}} \Big\rangle \Big |^2\\\leqslant& \Delta t^2\Vert \Lambda _x \Vert ^2 \Vert W \Vert ^2\Big (\sum \limits _{i,j} \Delta x\Delta y \Vert D_{i+\frac{1}{2},j}\Vert ^2+\sum \limits _{i,j}\Delta x \Delta y\Vert D_{i,j+\frac{1}{2}}\varphi \Vert ^2\Big )\\ {}&\times \Big (\sum \limits _{i,j} \Delta x\Delta y \Vert {\widehat{\textbf{F}}}_{i+\frac{1}{2},j}\Vert ^2 +\sum \limits _{i,j}\Delta x \Delta y\Vert {\widehat{\textbf{F}}}_{i,j+\frac{1}{2}}\Vert ^2 \Big ) \\\leqslant& \gamma ^2\Delta t^2\Vert \varphi \Vert _{H^1}^2\Vert \textbf{F}\Vert _{2,I} ^2. \end{aligned}$$

Then the proof is complete. 

Before we proceed to Proposition 1, we need a further result on the behaviour of $L_1$.

#### Lemma 2

Let us consider $\textbf{F},\textbf{F}'$ and $\textbf{G},\textbf{G}'$ such that

$$\begin{aligned}{}[L_1(\textbf{F})]_l=\textbf{G}_l,\quad [L_1(\textbf{F}')]_l=\textbf{G}'_l \end{aligned}$$

for some *l*. And we assume that there exist $\gamma ,\gamma '>0$ such that

$$\begin{aligned} \big\Vert \left(\text {Id}_{M\times M}+\frac{\Delta t}{\varepsilon }W\right)^{-1} \big\Vert \leqslant \gamma ,\quad \frac{\Delta t}{\varepsilon }\big\Vert \left(\text {Id}_{M\times M}+\frac{\Delta t}{\varepsilon }W\right)^{-1}W \big\Vert \leqslant \gamma ', \end{aligned}$$

then there exists a constant $\eta >0$, independent of $\textbf{F}, \textbf{F}', I$, and $\varepsilon$ such that

$$\begin{aligned} \Vert \textbf{F}_l- \textbf{F}'_l \Vert _{2,I}\leqslant \eta \Vert \textbf{G}_l- \textbf{G}'_l \Vert _{2,I}, \quad \Vert \textbf{F}_l- \textbf{F}'_l \Vert _{-1,I}\leqslant \eta \Vert \textbf{G}_l- \textbf{G}'_l \Vert _{-1,I}. \end{aligned}$$

#### Proof

To prove the lemma, we apply ${\mathbb {P}}$ to $[L_1(\textbf{F})]_l=\textbf{G}_l$, and we get

$$\begin{aligned} {\mathbb {P}}\textbf{F}_l={\mathbb {P}}\textbf{G}_l+{\mathbb {P}}\textbf{F}_l ^{(0)}-\frac{\Delta t}{\Delta x}{\mathbb {P}}B\Lambda _x\delta _l ^x \textbf{F}^{(0)}-\frac{\Delta t}{\Delta y}{\mathbb {P}}B\Lambda _y\delta _l ^y \textbf{F}^{(0)} .\end{aligned}$$

Now, we substitute ${\mathbb {P}}\textbf{F}$ into the Maxwellian in the equation $[L_1(\textbf{F})]_l=\textbf{G}_l$, we have

$$\begin{aligned} \textbf{F}_l=\,&\textbf{G}_l +\textbf{F}_l ^{(0)}-\frac{\Delta t}{\Delta x}B\Lambda _x\delta _l ^x \textbf{F}^{(0)}-\frac{\Delta t}{\Delta y}B\Lambda _y\delta _l ^y \textbf{F}^{(0)}\\&+\frac{\Delta t}{\varepsilon }W\Big [ {\mathbb {M}}\big ({\mathbb {P}}\textbf{G}_l+{\mathbb {P}}\textbf{F}_l ^{(0)}-\frac{\Delta t}{\Delta x}{\mathbb {P}}B\Lambda _x\delta _l ^x \textbf{F}^{(0)}-\frac{\Delta t}{\Delta y}{\mathbb {P}}B\Lambda _y\delta _l ^y \textbf{F}^{(0)}\big )-\textbf{F}_l\Big ]\\&+\frac{\Delta t}{\varepsilon }\textbf{w}_0\otimes \Big ( {\mathbb {M}}({\mathbb {P}}\textbf{f}_l ^{n,0})-\textbf{f}_l^{n,0}\Big ) .\end{aligned}$$

We can collect all the unknown terms $\textbf{F}_l$ on the left-hand side

$$\begin{aligned}\textbf{F}_l=&\left(\text {Id}_{M\times M}+\frac{\Delta t}{\varepsilon }W\right)^{-1} \Big [\textbf{G}_l +\textbf{F}_l ^{(0)}-\frac{\Delta t}{\Delta x}B\Lambda _x\delta _l ^x \textbf{F}^{(0)}-\frac{\Delta t}{\Delta y}B\Lambda _y\delta _l ^y \textbf{F}^{(0)}\\&+\frac{\Delta t}{\varepsilon }W {\mathbb {M}}\big ({\mathbb {P}}\textbf{G}_l+{\mathbb {P}}\textbf{F}_l ^{(0)}-\frac{\Delta t}{\Delta x}{\mathbb {P}}B\Lambda _x\delta _l ^x \textbf{F}^{(0)}-\frac{\Delta t}{\Delta y}{\mathbb {P}}B\Lambda _y\delta _l ^y \textbf{F}^{(0)}\big )\\&+\frac{\Delta t}{\varepsilon }\textbf{w}_0\otimes \big ( {\mathbb {M}}({\mathbb {P}}\textbf{f}_l ^{n,0})-\textbf{f}_l^{n,0}\big ) \Big ]. \end{aligned}$$

We have

$$\begin{aligned} \textbf{F}_l-\textbf{F}'_l= \left(\text {Id}_{M\times M}+\frac{\Delta t}{\varepsilon }W\right)^{-1} \Big (\textbf{G}_l-\textbf{G}'_l+\frac{\Delta t}{\varepsilon }W{\mathbb {M}}({\mathbb {P}}\textbf{G}_l-{\mathbb {P}}\textbf{G}'_l)\Big ). \end{aligned}$$

This concludes the proof. 

#### Proposition 1

Under the conditions of Lemmas 1 and 2, if $\textbf{F}^{*}$ is the unique solution of $L_2(\textbf{F})=0$, there exists $\theta$ independent of $\varepsilon$ such that we have

$$\begin{aligned} \Vert \textbf{F}^{(r+1)}- \textbf{F}^{*} \Vert _{L^2}\leqslant (\theta \Delta t)^{r+1} \Vert \textbf{F}^{(0)}- \textbf{F}^{*} \Vert _{L^2}. \end{aligned}$$

#### Proof

We have

$$\begin{aligned} L_1(\textbf{F}^{(r+1)})- L_1(\textbf{F}^{*})&=L_1(\textbf{F}^{(r)})-L_2(\textbf{F}^{(r)})- L_1(\textbf{F}^{*})\\&=\big (L_1(\textbf{F}^{(r)})- L_1(\textbf{F}^{*})\big )-\big ( L_2(\textbf{F}^{(r)})- L_2(\textbf{F}^{*}) \big ). \end{aligned}$$

Also, we can write a Poincaré inequality for $\varphi$ as follows:

$$\begin{aligned} \Vert \varphi \Vert _{2,I}\leqslant C\Vert D\varphi \Vert _{2,I}, \end{aligned}$$

where *C* is a constant. Using the results from the Poincaré inequality, Lemmas 1 and 2, the proof is concluded. 

Starting from the Chapman-Enskog expansion of (2a), we can show that our method is asymptotic preserving. We have

18 $$\begin{aligned} \left\{ \begin{aligned} &\textbf{f}={\mathbb {M}}(\textbf{u}^{\varepsilon })+\mathcal {O}(\varepsilon ), \\& \dfrac{\partial \textbf{u}^{\varepsilon }}{\partial t}+\dfrac{\partial \textbf{A}_1(\textbf{u}^{\varepsilon })}{\partial x}+\dfrac{\partial \textbf{A}_2(\textbf{u}^{\varepsilon })}{\partial y}=\mathcal {O}(\varepsilon ). \end{aligned} \right. \end{aligned}$$

#### Proposition 2

Equation (14a) is consistent with the limit model (18) up to $\mathcal {O}(\varepsilon )$.

#### Proof

Let us define $\textbf{u}^{\varepsilon ,(r)}={\mathbb {P}}\textbf{F}^{(r)}$, from (12) we get

$$\begin{aligned}{}  {} \textbf{u}^{\varepsilon ,(r+1)}=\,&{\mathbb {P}}\textbf{F}^{(0)}-\frac{\Delta t}{\Delta x}{\mathbb {P}}\Lambda _xW\delta ^x \textbf{F}^{(r)}-\frac{\Delta t}{\Delta x}\textbf{w}_0\otimes {\mathbb {P}} \Lambda _x\delta ^x \textbf{f}^{n,0}\\{} & {} -\frac{\Delta t}{\Delta y}{\mathbb {P}}\Lambda _yW\delta ^y \textbf{F}^{(r)}-\frac{\Delta t}{\Delta y}\textbf{w}_0\otimes {\mathbb {P}} \Lambda _y\delta ^y \textbf{f}^{n,0}+\mathcal {O}(\varepsilon ). \end{aligned}$$

Using (3), we have

$$\begin{aligned} \textbf{F}^{(r+1)}=\,&{\mathbb {M}}\big ({\mathbb {P}}\textbf{F}^{(0)}-\frac{\Delta t}{\Delta x}{\mathbb {P}}\Lambda _xW\delta ^x \textbf{F}^{(r)}-\frac{\Delta t}{\Delta x}\textbf{w}_0\otimes {\mathbb {P}} \Lambda _x\delta ^x \textbf{f}^{n,0}\\ {}&-\frac{\Delta t}{\Delta y}{\mathbb {P}}\Lambda _yW\delta ^y \textbf{F}^{(r)}-\frac{\Delta t}{\Delta y}\textbf{w}_0\otimes {\mathbb {P}} \Lambda _y\delta ^y \textbf{f}^{n,0}\big )+\mathcal {O}(\varepsilon )\\=\,&{\mathbb {M}}(\textbf{u}^{\varepsilon ,(r+1)})+\mathcal {O}(\varepsilon ). \end{aligned}$$

Then

$$\begin{aligned}\textbf{u}^{\varepsilon ,(r+1)}=\,&\textbf{u}^{\varepsilon ,(0)} -\frac{\Delta t}{\Delta x}W\delta ^x{\mathbb {P}}\Lambda _x \textbf{F}^{(r)}\\&-\frac{\Delta t}{\Delta x}\textbf{w}_0\otimes \delta ^{x}{\mathbb {P}}\Lambda _x \textbf{f}^{n,0}-\frac{\Delta t}{\Delta y}W\delta ^y{\mathbb {P}}\Lambda _y \textbf{F}^{(r)}-\frac{\Delta t}{\Delta y}\textbf{w}_0\otimes \delta ^y{\mathbb {P}} \Lambda _y \textbf{f}^{n,0}+\mathcal {O}(\varepsilon ) \\=\,&\textbf{u}^{\varepsilon ,(0)} -\frac{\Delta t}{\Delta x}W\delta ^x\textbf{A}_1(\textbf{u}^{\varepsilon ,(r)})-\frac{\Delta t}{\Delta x}\textbf{w}_0\otimes \delta ^{x}\textbf{A}_1(\textbf{u}^{\varepsilon ,(0)})-\frac{\Delta t}{\Delta y}W\delta ^y\textbf{A}_2(\textbf{u}^{\varepsilon ,(r)})\\ {}&-\frac{\Delta t}{\Delta y}\textbf{w}_0\otimes \delta ^{y}\textbf{A}_2(\textbf{u}^{\varepsilon ,(0)})+\mathcal {O}(\varepsilon ). \end{aligned}$$

We observe that if the the spatial discretisation is consistent with the derivative in space, the above formula is the space discretisation and the time of the asymptotic model (18), and the result is concluded. 

### Time Discretisation in the $L_2$ Operator

We consider the first, second, and fourth order approximations in time in the $L_2$ operator, when there is no source term.

(i) For the first order approximation, we get
  $$\begin{aligned} {\mathbb {P}}\textbf{f}^{n,1}-{\mathbb {P}}\textbf{f}^{n,0}+\frac{\Delta t}{\Delta x}{\mathbb {P}}(\Lambda _x\delta ^x\textbf{f}^{n,1})+\frac{\Delta t}{\Delta y}\Lambda _y{\mathbb {P}}(\delta ^y\textbf{f}^{n,1})=0, \end{aligned}$$
  where $\textbf{f}^{n,0}=\textbf{f}^n$ and $\textbf{f}^{n,1}\approx \textbf{f}(t_{n+1})$. The matrix *W* becomes $W=(1)$. Then, we get
  $$\begin{aligned} \left(\text {Id}_{1\times 1}+\frac{\Delta t}{\varepsilon }W\right)^{-1}=\frac{\varepsilon }{\varepsilon +\Delta t},\quad \frac{\Delta t}{\varepsilon }\left(\text {Id}_{1\times 1}+\frac{\Delta t}{\varepsilon }W\right)^{-1}W=\frac{\Delta t}{\varepsilon +\Delta t}. \end{aligned}$$
  We observe that these two matrices are uniformly bounded.
(ii) For the second order approximation, which is the Crank-Nicholson method, the scheme becomes
  $$\begin{aligned}{} & {} {\mathbb {P}} \textbf{f}^{n,1}-{\mathbb {P}}\textbf{f}^{n,0}+\frac{\Delta t}{\Delta x}\big (\frac{1}{2}{\mathbb {P}}(\Lambda _x\delta ^x\textbf{f}^{n,0})+\frac{1}{2}{\mathbb {P}}(\Lambda _x\delta ^x\textbf{f}^{n,1})\big )\\{} & {} +\frac{\Delta t}{\Delta y}\big (\frac{1}{2}{\mathbb {P}}(\Lambda _y\delta ^y\textbf{f}^{n,0})+\frac{1}{2}{\mathbb {P}}(\Lambda _y\delta ^y\textbf{f}^{n,1})\big )=0, \end{aligned}$$
  also we have $W=(\frac{1}{2})$. Similarly, we see that two matrices $(\text {Id}_{1\times 1}+\frac{\Delta t}{\varepsilon }W)^{-1}$ and $\frac{\Delta t}{\varepsilon }(\text {Id}_{1\times 1}+\frac{\Delta t}{\varepsilon }W)^{-1}W$ are uniformly bounded.
(iii) For the fourth-order scheme [16], we get
  $$\begin{aligned} &{\mathbb {P}}\textbf{f}^{n,1}-{\mathbb {P}}\textbf{f}^{n,0}+\frac{\Delta t}{\Delta x}\big (\frac{5}{24}{\mathbb {P}}(\Lambda _x\delta ^x\textbf{f}^{n,0})+\frac{1}{3}{\mathbb {P}}(\Lambda _x\delta ^x\textbf{f}^{n,1})-\frac{1}{24}{\mathbb {P}}(\Lambda _x\delta ^x\textbf{f}^{n,2})\big )\\ {}&+\frac{\Delta t}{\Delta y}\big (\frac{5}{24}{\mathbb {P}}(\Lambda _y\delta ^y\textbf{f}^{n,0})+\frac{1}{3}{\mathbb {P}}(\Lambda _y\delta ^y\textbf{f}^{n,1})-\frac{1}{24}{\mathbb {P}}(\Lambda _y\delta ^y\textbf{f}^{n,2})\big )=0, \\ &{\mathbb {P}} \textbf{f}^{n,2}-{\mathbb {P}}\textbf{f}^{n,0}+\frac{\Delta t}{\Delta x}\big (\frac{1}{6}{\mathbb {P}}(\Lambda _x\delta ^x\textbf{f}^{n,0})+\frac{2}{3}{\mathbb {P}}(\Lambda _x\delta ^x\textbf{f}^{n,1})+\frac{1}{6}{\mathbb {P}}(\Lambda _x\delta ^x\textbf{f}^{n,2})\big )\\ {}&+\frac{\Delta t}{\Delta y}\big (\frac{1}{6}{\mathbb {P}}(\Lambda _y\delta ^y\textbf{f}^{n,0})+\frac{2}{3}{\mathbb {P}}(\Lambda _y\delta ^y\textbf{f}^{n,1})+\frac{1}{6}{\mathbb {P}}(\Lambda _y\delta ^y\textbf{f}^{n,2})\big )=0, \end{aligned}$$
  where $\textbf{f}^{n,0}=\textbf{f}^n$, $\textbf{f}^{n,1}\approx \textbf{f}(t_n+\frac{\Delta t}{2})$, and $\textbf{f}^{n,2}\approx \textbf{f}(t_{n+1})$. Also, we have
  $$\begin{aligned} W=\begin{pmatrix} \frac{1}{3}&{} -\frac{1}{24} \\ \frac{2}{3} &{} \frac{1}{6} \end{pmatrix}. \end{aligned}$$
  It is easy to observe that the matrix $\text {Id}_{2\times 2}+\frac{\Delta t}{\varepsilon }W$ is invertible and the matrices $(\text {Id}_{2\times 2}+\frac{\Delta t}{\varepsilon }W)^{-1}$ and $\frac{\Delta t}{\varepsilon }(\text {Id}_{2\times 2}+\frac{\Delta t}{\varepsilon }W)^{-1}W$ are uniformly bounded.

## Space Discretisation

### Definition of the $\delta ^x$ and $\delta ^y$ Operators

Since $\Lambda _x$ and $\Lambda _y$ are diagonal matrices, we can consider the scalar transport equation

$$\begin{aligned} \dfrac{\partial f}{\partial t}+a\dfrac{\partial f}{\partial x}+b\dfrac{\partial f}{\partial y}=0, \end{aligned}$$

where *a* and *b* are constants and both of them can not be zero at the same time. Next, we will discuss the approximation of $\dfrac{\partial f_l}{\partial x}$, and the approximation for $\dfrac{\partial f_l}{\partial y}$ is obtained in a similar manner. [17] has developed stable numerical finite difference methods for first-order hyperbolics in one dimension, which use *s* forward and *r* backward steps in the discretisation of the space derivatives that are of order at most $2\min \{r+1,s\}$. These methods are based on the approximation of $\dfrac{\partial f}{\partial x}(x_i,y_j),$, $i,j \in {\mathbb {Z}}$, by a finite difference

$$\begin{aligned} \frac{1}{\Delta x} \sum \limits _{k=-r}^{s}\alpha _kf_{i+k,j} ,\end{aligned}$$

when $a>0$ and we say that the method is of the class $\{r,s\}$. If $a<0$, we set

$$\begin{aligned} \frac{1}{\Delta x} \sum \limits _{k=-s}^{r}\alpha _{-k}f_{i+k,j}. \end{aligned}$$

Throughout this section we suppose without loss of generality that $a > 0$. Otherwise, the roles of *r* and *s* are reversed. We call an $\{r, s\}$ method of the highest order an interpolatory method, and it is denoted by [*r*.*s*]. Following the theorem of [17], for all integers $r,s\geqslant 0,$ the order of the interpolatory method [*r*.*s*] is $q=r+s$, i.e.,

$$\begin{aligned} \frac{\delta ^x f_{i,j}}{\Delta x}-\dfrac{\partial f}{\partial x}(x_i,y_j) =c_{r,s}(\Delta x)^q\frac{\partial ^{q+1}f}{\partial x^{q+1}}(x_i,y_j)+\mathcal {O} ((\Delta x)^{q+1}), \end{aligned}$$

where the error constant $c_{r,s}$ is defined by

$$\begin{aligned} c_{r,s}=\frac{(-1)^{s-1}r!s!}{(r+s+1)!}. \end{aligned}$$

The coefficients are defined by

$$\begin{aligned} \alpha _k&=\frac{(-1)^{k+1}}{k} \frac{r!s!}{(r+k)!(s-k)!},\quad -r\leqslant k\leqslant s,\quad k\ne 0,\\ \alpha _0&=-\sum \limits _{k=-r,k\ne 0}^s \alpha _k. \end{aligned}$$

We recall that the [*r*, *r*], $[r, r+1],$ and $[r, r+2]$ schemes are the only stable interpolatory methods, and we will only consider these approximations. Before we proceed to the possible choices for $\delta$, it is important to remark that, we can write

$$\begin{aligned} \delta ^x f_{i,j}= {\hat{f}}_{i+\frac{1}{2},j}-{\hat{f}}_{i-\frac{1}{2},j}, \end{aligned}$$

where

$$\begin{aligned} {\hat{f}}_{i+\frac{1}{2},j}=\sum \limits _{k=-s+1}^{r} \beta _k f_{i+k,j} \end{aligned}$$

with $\beta _k=\sum \limits _{m\geqslant k} \alpha _m$. In the following, we consider the first, second, and fourth order approximations in *x*, that in *y* are done in a similar manner.

(i) First order. Here, we must have $r=0$, $s=1$. We get the upwind scheme
  $$\begin{aligned} \delta _1 ^x f_{i,j}=f_{i,j}-f_{i-1,j}. \end{aligned}$$
  Then, we have
  $$\begin{aligned} \dfrac{\partial f}{\partial x}(x_i,y_j)=\frac{1}{\Delta x}(f_{i,j}-f_{i-1,j}) +c_{0,1}\Delta x\frac{\partial ^{2}f}{\partial x^{2}}(x_i,y_j)+\mathcal {O} ((\Delta x)^{2}), \end{aligned}$$
  and the flux is given by
  $$\begin{aligned} {\hat{f}}_{i+\frac{1}{2},j} =f_{i+1,j}. \end{aligned}$$
(ii) Second order case. Two cases are possible.
  $$r=s=1$$: centered case
    $$\begin{aligned} \delta _2^xf_{i,j}=\frac{1}{2}\big ( f_{i+1,j}-f_{i-1,j}\big ) \end{aligned}$$
    and the flux is
    $$\begin{aligned} {\hat{f}}_{i+1/2,j}=\frac{f_{i+1,j}+f_{i,j}}{2}. \end{aligned}$$
  $$r=0$$, $$s=2$$. There
    $$\begin{aligned} \delta _2^xf=\frac{3}{2}f_{i,j}-2f_{i-1,j}+\frac{1}{2}f_{i-2,j}, \end{aligned}$$
    and the flux is
    $$\begin{aligned} {\hat{f}}_{i+1/2,j}=\frac{3}{2}f_{i,j}-\frac{1}{2}f_{i-1,j}. \end{aligned}$$
  The centered case will no longer be considered.
(iii) Third order. Only choice is possible $r=1$, $s=2$ and we get
  $$\begin{aligned} \delta _3 ^x f_{i,j}=\frac{f_{i-2,j}}{6}-f_{i-1,j}+\frac{f_{i,j}}{2}+\frac{f_{i+1,j}}{3}. \end{aligned}$$
  Therefore, we have
  $$\begin{aligned}{}  {} \dfrac{\partial f}{\partial x}(x_i,y_j)=&\frac{1}{\Delta x}\left(\frac{f_{i-2,j}}{6}-f_{i-1,j}+\frac{f_{i,j}}{2}+\frac{f_{i+1,j}}{3}\right)\\{} & {} +c_{2,1}(\Delta x)^3\frac{\partial ^{4}f}{\partial x^{4}}(x_i,y_j)+\mathcal {O} ((\Delta x)^{4}). \end{aligned}$$
  As before, the flux is given by
  $$\begin{aligned} {\hat{f}}_{i+\frac{1}{2},j} =-\frac{f_{i-1,j}}{6}+\frac{5}{6}f_{i,j} +\frac{f_{i+1,j}}{3}. \end{aligned}$$
(iv) Fourth order. We consider two cases.
  If $r=s=2$, we have
    $$\begin{aligned} \delta _4 ^x f_{i,j}=\frac{f_{i-2,j}}{12}-\frac{2}{3}f_{i-1,j}+\frac{2}{3}f_{i+1,j}-\frac{f_{i+2,j}}{12}. \end{aligned}$$
    We can write
    $$\begin{aligned}{} & {} \dfrac{\partial f}{\partial x}(x_i,y_j)=\frac{1}{\Delta x}\left(\frac{f_{i-2,j}}{12} -\frac{2}{3}f_{i-1,j}+\frac{2}{3}f_{i+1,j}-\frac{f_{i+2,j}}{12}\right) +c_{2,2}(\Delta x)^4\frac{\partial ^{5}f}{\partial x^{5}}(x_i,y_j)+\mathcal {O} ((\Delta x)^{5}). \end{aligned}$$
    For the flux, we can write as follows:
    $$\begin{aligned} {\hat{f}}_{i+\frac{1}{2},j} =\frac{f_{i-1,j}}{12}+\frac{3}{4}f_{i,j} +\frac{3}{4}f_{i+1,j}+\frac{f_{i+2,j}}{12}. \end{aligned}$$
  If $r=1$ and $s=3$, we have
    $$\begin{aligned} \delta _4 ^x f_{i,j}=-\frac{f_{i-3,j}}{12}+\frac{f_{i-2,j}}{2}-\frac{3}{2}f_{i-1,j}+\frac{5}{6}f_{i,j}+\frac{f_{i+1,j}}{4}. \end{aligned}$$
    Hence
    $$\begin{aligned}{}  {} \dfrac{\partial f}{\partial x}(x_i,y_j)=&\frac{1}{\Delta x}\left(-\frac{f_{i-3,j}}{12}+\frac{f_{i-2,j}}{2}-\frac{3}{2}f_{i-1,j}+\frac{5}{6}f_{i,j}+\frac{f_{i+1,j}}{4}\right)\\{} & {} +c_{1,3}(\Delta x)^4\frac{\partial ^{5}f}{\partial x^{5}}(x_i,y_j)+\mathcal {O} ((\Delta x)^{5}). \end{aligned}$$
    The flux becomes
    $$\begin{aligned} {\hat{f}}_{i+\frac{1}{2},j} =\frac{f_{i-2,j}}{12}-\frac{5}{12}f_{i-1,j} +\frac{13}{12}f_{i,j}+\frac{f_{i+1,j}}{4}. \end{aligned}$$
    We will only use the case $r=1$, $s=3$ because the case $r=2$, $s=2$ is centered.

### Limitation

We have explored two ways to introduce a nonlinear stabilisation mechanism. The first one was inspired from [21, 24, 26] and consists in “limiting” the flux, and the second one was inspired by the MOOD paradigm [14, 25].

#### Limitation of the Flux

In this section, we only consider the space discretisation in *x*, that in *y* is done in a similar manner. First, we calculate the difference between the first and the second order approximations in *x*. We have

$$\begin{aligned} \delta _2 ^x f_{i,j}-\delta _1 ^x f_{i,j}=\frac{1}{6}(f_{i-2,j}-3f_{i,j}+2f_{i+1,j}). \end{aligned}$$

So for limitation

$$\begin{aligned}\widetilde{\delta _2^x} f_{i,j}&=\delta _1 ^x f_{i,j}+\frac{\theta }{6}(\delta _2 ^x f_{i,j}-\delta _1 ^x f_{i,j})\\&=\delta _1 ^x f_{i,j}+\frac{\theta }{6}(f_{i-2,j}-3f_{i,j}+2f_{i+1,j})\\&=\delta _1 ^x f_{i,j} \left(1+\frac{\theta r}{6}\right), \end{aligned}$$

where $$\theta \in [0,1]$$ and

$$\begin{aligned} r=\frac{f_{i-2,j}-3f_{i,j}+2f_{i+1,j}}{f_{i,j}-f_{i-1,j}} =1-\frac{-2f_{i+1,j}+4f_{i,j}-f_{i-1,j}-f_{i-2,j}}{f_{i,j}-f_{i-1,j}}. \end{aligned}$$

We can see that if *f* is smooth, $r \approx 0$. To have a monotonicity condition, we also need

$$\begin{aligned} 6+\theta r \geqslant 0. \end{aligned}$$

We want to find $\theta (r)$ such that $\theta (0)=1$ and

$$\begin{aligned} 0\leqslant 1+\frac{\theta (r)r}{6} \leqslant M, \end{aligned}$$

where *M* is a constant that will dictate the CFL constraint. Since $\theta (0)=1$, we observe that $M\geqslant 1$ is a necessary condition. One solution is to choose a $\alpha \in ]0,\min \{6,6(M-1) \}[$. We take

$$\begin{aligned} \theta (r) = {\left\{ \begin{array}{ll} 1 &{} \text {if }r \in [-6+\alpha ,6(M-1)-\alpha ], \\ \frac{-6+\alpha }{r} &{} \text {if }r <-6+\theta , \\ \frac{6(M-1)-\alpha }{r} &{}\text {if } r> 6(M-1)+\alpha . \end{array}\right. } \end{aligned}$$

After calculations, we get

$$\begin{aligned} \widetilde{\delta _2 ^x}=\delta _1 ^x (1+\theta r)=\delta _1 ^x+\psi (\delta _1 ^x,\delta _2 ^x)(\delta _2 ^x-\delta _1 ^x), \end{aligned}$$

where

$$\begin{aligned} \psi (r,s)(s-r) = {\left\{ \begin{array}{ll} s-r &{} \text {if }\frac{s}{r} \in [-6+\alpha ,6(M-1)-\alpha ], \\ (-6+\alpha ) r &{} \text {if }\frac{s}{r} <-6+\theta , \\ (6(M-1)-\alpha ) r &{} \text {if } \frac{s}{r}> 6(M-1)+\alpha . \end{array}\right. } \end{aligned}$$

For the first and fourth order approximations, it is possible to follow the same technique.

The main drawback of this approach is that the projection on the conservative variables plays no role, while the variables we are really interested in are these variables, so we can expect some weaknesses.

#### Stabilisation by the MOOD Technique

This was inspired from [14, 25], the variables on which we test are the physical variables $(\rho , \textbf{v}, p)$. This section also justifies the way that have written the update of the spatial term as in (15), i.e., a sum of residual that are evaluated on the elements $K_{i+1/2.j+1/2}=[x_i,x_{i+1}]\times [y_j, y_{j+1}]$.

Starting from $\textbf{F}^n$, we first compute $\widetilde{\textbf{F}^{n+1}}$ with the full order method (i.e., full order in time and space). In the numerical examples, we will take the fourth order accurate method, but other choices can be made. The algorithm is as follow: we define a vector of logical $\texttt {Flag}_p$ which is false initially for all the grid points, and a vector of logical $\texttt {Flag}_e$ which is also set to false.

(i) For each mesh point $(x_i,y_j)$, we compute ${\tilde{V}}_{ij}=\widetilde{(\rho , \textbf{v}, p)}^{n+1}$ variables defined by $\mathbb P\widetilde{\textbf{F}^{n+1}}$. If $\tilde{\rho }^{n+1}_{ij}\leqslant 0$ or $\tilde{p}^{n+1}_{ij}\leqslant 0$ or one of the components of ${\tilde{V}}_{ij}$ are NaN,^1^ we set $\texttt {Flag}_p(i,j)=.true.$
(ii) Then we loop over the quads $[x_i,x_{i+1}]\times [y_j, y_{j+1}]$. If for one of the four corners $(x_l,y_q)$, $\texttt {Flag}_p(l,q)=.true.$, we set $\texttt {Flag}_e(i+1/2,j+1/2)=.true.$
(iii) For each element such that $\texttt {Flag}_e(i+1/2,j+1/2)=.true.$, we recompute, for each sub-time step the four residuals defined by (15b).

In [14, 25], there is also a way to detect local extremes, and in [25] to differentiate the local smooth extremes from the discontinuities. We have not used this here, and there is a way to improve. The first-order version of our scheme amounts to global Lax-Friedrichs. To make sure that the first-order scheme is domain invariant preserving, we can apply this procedure to each of the cycles of the DeC procedure, we have not done that in the numerical examples.

## Stability Analysis

We will study the stability of the discretisation of the homogeneous problem. As discussed in the previous section, since the matrices $\Lambda _x$ and $\Lambda _y$ are diagonal, it is enough to consider again the following transport equation:

19 $$\begin{aligned} \dfrac{\partial f}{\partial t} +a\dfrac{\partial f}{\partial x} +b\dfrac{\partial f}{\partial y}=0. \end{aligned}$$

Since $ab=0$ in the case of a four-wave model, we have the same results as the one-dimensional case [3]. In other cases, we perform the Fourier analysis to evaluate the amplification factors of the method, first without defect correction iteration, then with defect correction iteration. We assume, without loss of generality, that $a,b>0$. we denote the Fourier symbol of $\delta ^x$ and $\delta ^y$ as $g_1$ and $g_2$, respectively. For $\delta ^x$ and $\delta ^y$ operators, we considered four cases in the previous section, we have

- First order in both *x* and *y*: we have $g^{(1)}(\theta )=1-\text{e}^{i\theta }$ and $g_1=g^{(1)}(\theta _1)$, $g_2=g^{(1)}(\theta _2).$ We see that $\Re (g^{(1)})\geqslant 0$ and $\max _\theta \vert g^{(1)}\vert = 4$.
- Second order in *x* and *y*: $g_1=g^{(2)}(\theta _1)$ and $g_2=g^{(2)}(\theta _2),$ where
  $$\begin{aligned} g^{(2)}(\theta )=\frac{3}{2}-2\text{e}^{-i\theta }+\frac{\text{e}^{-2i\theta }}{2}. \end{aligned}$$
  We notice that $\Re (g^{(2)})=\big ( \cos \theta -1\big )^2\geqslant 0$ and $\max _\theta \vert g^{(2)}\vert = 2$.
- Third order in both *x* and *y*: $g_1=g^{(3)}(\theta _1)$ and $g_2=g^{(3)}(\theta _2),$ where
  $$\begin{aligned} g^{(3)}=\frac{\text{e}^{-2i\theta }}{6}-\text{e}^{-i\theta }+\frac{1}{2}+\frac{\text{e}^{i\theta }}{3}. \end{aligned}$$
  Again, $$\Re (g^{(3)})=\frac{1}{3}\big (\cos \theta -1\big )^2\geqslant 0$$ and $$\max _\theta \vert g^{(3)}\vert = \tfrac{3}{2}$$.
- Fourth order in both *x* and *y*: we only consider the case $r=1$, $s=2$. We have $g_1=g^{(4)}(\theta )$ and $g_2=g^{(4)}(\theta _2),$ where
  $$\begin{aligned} g^{(4)}(\theta -1)=-\frac{\text{e}^{-3i\theta }}{12}+\frac{\text{e}^{-2i\theta }}{2}-\frac{3}{2}\text{e}^{-i\theta }+\frac{5}{6}+\frac{\text{e}^{i\theta }}{4}, \end{aligned}$$
  and we have $\Re (g^{(4)})=\frac{1}{3}\big (1-\cos \theta \big )^3\geqslant 0$ and $\max _\theta \vert g^{(4)}\vert = \frac{8}{3}.$

Now, we consider the first, second, and fourth order approximations in time in the $L_2$ operator. In the sequel, we set $g=\mu g_1+\nu g_2$ with $\mu =a\frac{\Delta t}{\Delta x}$ and $\nu =b\frac{\Delta t}{\Delta y}$.

(i) First order in time. Using the Fourier transformation, the $L_2$ operator can be written as
  $$\begin{aligned} {\hat{f}}^{n+1} -{\hat{f}}^{n}+\mu g_1 {\hat{f}}^{n+1}+\nu g_2 {\hat{f}}^{n+1}=0. \end{aligned}$$
  The amplification factor is
  $$\begin{aligned} G_1=\frac{1}{1+g}. \end{aligned}$$
  In order to have a stable scheme for the first-order scheme, we should have $|G|\leqslant 1$, and a necessary and sufficient condition is $2\Re (g)+|g|^2\geqslant0$. The defect correction iteration is written as
  $$\begin{aligned} {\hat{f}}^{(r+1)} ={\hat{f}}^{n}-\mu g_1 {\hat{f}}^{(r)}-\nu g_2 {\hat{f}}^{(r)} .\end{aligned}$$
  The resulting formula for the amplification factor $G_{r+1},$ is given by
  $$\begin{aligned} G_{1,0}&=1 ,\\ G_{1, r+1}(g)&=1-g G_{1,r}(g). \end{aligned}$$
  We can observe that
  20 $$\begin{aligned} G_{1,r+1}(g)-G_1(g)= (-1)^{r+1}g^{r+1} \big (1-G_1(g)\big ). \end{aligned}$$
  We note that $g^{r+1}\rightarrow 0$ if $\vert g\vert <1$.
(ii) Second order in time. We again use the Fourier transformation, and write $L_2$ as
  $$\begin{aligned} {\hat{f}}^{n+1} -{\hat{f}}^{n}+\frac{\mu }{2}( g_1 {\hat{f}}^{n}+g_1 {\hat{f}}^{n+1})+\frac{\nu }{2} (g_2 {\hat{f}}^{n}+g_2 {\hat{f}}^{n+1})=0. \end{aligned}$$
  In this case the amplification factor is
  $$\begin{aligned} G_2=\frac{1-\frac{g }{2}}{1+\frac{g }{2} }. \end{aligned}$$
  We conclude that under the following condition stability holds:
  $$\begin{aligned} \text {Re}(g)\geqslant 0. \end{aligned}$$
  The defect correction iteration reads
  $$\begin{aligned} {\hat{f}}^{(r+1)}= {\hat{f}}^{n}-\frac{\mu }{2}( g_1 {\hat{f}}^{n}+g_1 {\hat{f}}^{(r)})-\frac{\nu }{2} (g_2 {\hat{f}}^{n}+g_2 {\hat{f}}^{(r)}), \end{aligned}$$
  we have
  $$\begin{aligned} G_{2,0}&=1 ,\\ G_{2,r+1}(g)&=1-\frac{g}{2}-\frac{g}{2}G_{2,r}(g). \end{aligned}$$
  It is easy to check that
  21 $$\begin{aligned} G_{2,r+1}(g)-G_3(g)= (-1)^{r+1}\big (\frac{g}{2} \big )^{r+1} \big (1-G_2(g)\big ). \end{aligned}$$
  We note that $\big (\frac{g}{2} \big )^{r+1}\rightarrow 0$ if $\vert g\vert g\leqslant 2$.
(iii) Fourth order in time. Similarly, we have the following formula for the $L_2$ operator:
  22 $$\begin{aligned}\left\{ \begin{aligned}&{\hat{f}}^{n+\frac{1}{2}}-{\hat{f}}^{n}+\mu \left(\frac{5}{24}g_1 {\hat{f}}^{n}+\frac{1}{3}g_1 {\hat{f}}^{n+\frac{1}{2}}-\frac{1}{24}g_1 {\hat{f}}^{n+1}\right)+\nu \left(\frac{5}{24}g_2 {\hat{f}}^{n}+\frac{1}{3}g_2 {\hat{f}}^{n+\frac{1}{2}}-\frac{1}{24}g_2 {\hat{f}}^{n+1}\right)=0,\\&{\hat{f}}^{n+1}-{\hat{f}}^{n}+\mu \left(\frac{1}{6}g_1 {\hat{f}}^{n}+\frac{2}{3}g_1 {\hat{f}}^{n+\frac{1}{2}}+\frac{1}{6}g_1 {\hat{f}}^{n+1}\right)+\nu \left(\frac{1}{6}g_2 {\hat{f}}^{n}+\frac{2}{3}g_2 {\hat{f}}^{n+\frac{1}{2}}+\frac{1}{6}g_2 {\hat{f}}^{n+1}\right)=0. \end{aligned}\right. \end{aligned}$$
  We can rewrite (22) in the matrix form
  $$\begin{aligned} \begin{pmatrix} {\hat{f}}^{n+\frac{1}{2}} \\ {\hat{f}}^{n+1} \end{pmatrix}=G_4\begin{pmatrix} {\hat{f}}^{n}\\ {\hat{f}}^{n} \end{pmatrix}, \end{aligned}$$
  where setting $$\theta =24+12g+2g^2$$
  $$\begin{aligned} \begin{aligned} G_4(g)&= \begin{pmatrix} 1 +\frac{g }{3} &{}-\frac{g }{24}\\ \frac{2g }{3} &{} 1 +\frac{g }{6} \end{pmatrix}^{-1}\begin{pmatrix} 1-\frac{5g }{24}\\ 1-\frac{g}{6} \end{pmatrix}\\& =\frac{1}{2\theta } \begin{pmatrix} {24-g^2}\\ {24-12g+2g^2}\\ \end{pmatrix} .\end{aligned} \end{aligned}$$
  In order to have a stable scheme, one should have $\max \{|G_1|,|G_2|\}\leqslant1$. The defect correction iteration reads
  23 $$\begin{aligned} \left\{\begin{aligned} {\hat{h}}_1^{(r+1)}&={\hat{f}}^{n}-\mu \left(\frac{5}{24}g_1 {\hat{f}}^{n}+\frac{1}{3}g_1 {\hat{h}}_1^{(r)}-\frac{1}{24}g_1 {\hat{h}}_2^{(r)}\right)-\nu \left(\frac{5}{24}g_2 {\hat{f}}^{n}+\frac{1}{3}g_2 {\hat{h}}_1^{(r)}-\frac{1}{24}g_2 {\hat{h}}_2^{(r)}\right),\\ {\hat{h}}_2^{(r+1)}&={\hat{f}}^{n}-\mu \left(\frac{1}{6}g_1 {\hat{f}}^{n}+\frac{2}{3}g_1 {\hat{h}}_1^{(r)}+\frac{1}{6}g_1 {\hat{h}}_2^{(r)}\right)-\nu \left(\frac{1}{6}g_2 {\hat{f}}^{n}+\frac{2}{3}g_2 {\hat{h}}_1^{(r)}+\frac{1}{6}g_2 {\hat{h}}_2^{(r)}\right). \end{aligned}\right. \end{aligned}$$
  Rewriting (23) in the matrix form, we obtain
  $$\begin{aligned} {\hat{h}}^{(r+1)}=\begin{pmatrix} 1- \frac{5g}{24}\\ 1- \frac{g }{6} \end{pmatrix} -gM {\hat{h}}^{(r)}, \end{aligned}$$
  where $$M=\begin{pmatrix} \frac{1}{3} &{} \frac{-1}{24}\\ \frac{2}{3} &{} \frac{1}{6} \end{pmatrix}$$. We get
  $$\begin{aligned} G_{4,0}&=\begin{pmatrix} 1 \\ 1 \end{pmatrix} ,\\ G_{4,r+1}(g)&=\begin{pmatrix} 1- \frac{5g }{24}\\ 1- \frac{g }{6} \end{pmatrix} -gM G_{4,r}(g). \end{aligned}$$
  Hence
  24 $$\begin{aligned} G_{4,r+1}(g)-G_4(g)= (-1)^{r+1}(g)^{r+1} M^{r+1} \Bigg (\begin{pmatrix} 1 \\ 1 \end{pmatrix}-G_4(g) \Bigg ). \end{aligned}$$
  We note that $(-1)^{r+1}(g)^{r+1} M^{r+1}\rightarrow 0$ if $\vert g\vert \Vert M\Vert _2<1$, i.e., if $\vert g\vert \leqslant \big (\tfrac{337+\sqrt{104\,353}}{1152}\big )^{-1}\approx 1.745\,356\,304$.

**Fig. 2:**
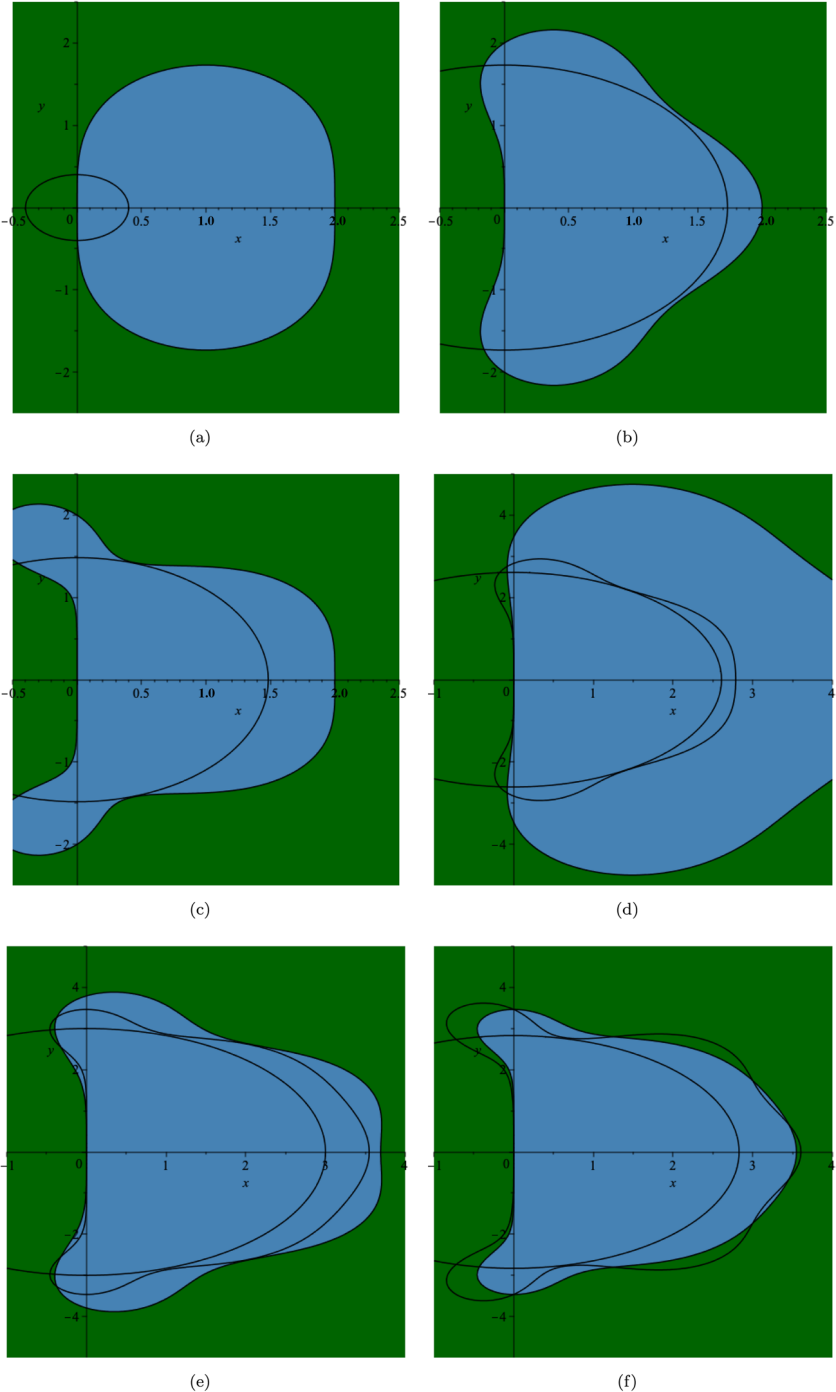
Plots of the stability region with the circle of radius $\rho$ for DeC second order (a) $\rho _2^2=0.4$, 2 iterations, (b) $\rho _3^2=\sqrt{3}$, 3 iterations, (c) $\rho _4^2=\sqrt{2.2}$, 4 iterations, and DeC fourth order (d) $\rho _4^4=\sqrt{6.8}$, 4 iterations, (e) $\rho _5^3=3$, 5 iterations, (f) $\rho _6^3=2\sqrt{2}$, 6 iterations

Using this relations, we have plotted the zone $\mathcal {A}_G$ where $\vert G\vert \leqslant 1$ for the second-order scheme (Crank Nicholson with DeC iteration) and $\max (\vert G(1)\vert , \vert G(2)\vert ) <1$. To get a stable scheme, we need that part of $\mathcal {A}_G$ has a non-empty intersection with $\{(x,y), x\geqslant 0\}$. We have plotted in Fig. 2$\mathcal {A}_G$ for the second-order Dec with 3 and 4 iterations, and for the fourth-order DeC with 4 and 5 iterations.

We see that the larger $\rho$ is achieved for $r+1$ iterations for scheme of order *r*.

From (20)–(24), the amplification factor of the two-dimensional scheme is $G_k^r\big (\nu g(\theta _1)+\mu g(\theta _2)\big )$, so we need to check when $\vert \nu g(\theta _1)+\mu g(\theta _2)\vert \leqslant \rho _k^r$. Since $\vert \nu g(\theta _1)+\mu g(\theta _2)\vert \leqslant 2 \max (|\mu |, |\nu |)\max _\theta \vert g(\theta )\vert$, we display $\max (|\mu |, |\nu |)$ in Table 1.

**Table 1:** CFL condition for the scheme DeC(*r*, *p*): *r* is the order, *p* is the number of iteraion

	DeC(2, 2)	DeC(2, 3)	DeC(2, 4)
$g^{(2)}$	0.4	$2/\sqrt{3}\approx 1.15$	$\sqrt{2.2}\approx 1.58$

	DeC(4,4)	DeC(4,5)	DeC(4,6)
$g^{(4)}$	$0.75\sqrt{6.8}\approx 1.95$	2.25	$1.5\sqrt{2}\approx 2.12$

Using these amplification factors, we obtain the maximum values of the CFL number for which the various options lead to the stability. Even with the BGK source term, the schemes are always stable for $\text{CFL}=1+\delta$, $\delta >0$. For example, the combination of fourth order in time/fourth order in space is stable for $\text{CFL}\approx 1.3$, and this can be checked experimentally on the vortex case below, with periodic boundary conditions. In that case, we have not been able to run higher than CFL 1.3, it may be an effect of the BGK source term. These results are also consistent with [3].

## Test Cases

In this section, we will present the numerical results that illustrate the behavior of our scheme on scalar problems and Euler equations. To evaluate the accuracy and robustness of the proposed high order asymptotic preserving kinetic scheme, in the following we perform the convergence analysis for the scalar problems and study several benchmark problems for the Euler equations of gas dynamics.

### Scalar Problems

Consider the two-dimensional advection equation:

$$\begin{aligned} \frac{\partial u}{\partial t}+ \dfrac{\partial u}{\partial x}+ \dfrac{\partial u}{\partial y} =0,~ (x,y,t)\in [-2,2]\times [-2,2]\times {\mathbb {R}}^{+}, \end{aligned}$$

and periodic boundary conditions. We consider the following initial condition:

$$\begin{aligned} u_0(x,y)=\sin (\uppi x+\uppi y),~ (x,y) \in (-2,2) \times (-2,2). \end{aligned}$$

The CFL number is set to 1. The convergence for the density is shown in Tables 2 and 3 for the final time $T = 10$ for orders 2 and 4, which result in the predicted convergence rates of second and fourth orders, respectively.

**Table 2:** Convergence study for the advection equation for order 2 at $T=10$

*h*	$L^1$-error	Slope	$L^2$-error	Slope	$L^{\infty }$-error	Slope
0.05	$1.069\,8\times10^{+1}$	–	$2.847\,9\times10^{0}$	–	$9.387\,8\times10^{-1}$	–
0.025	$3.559\,5\times10^{0}$	1.59	$9.621\,2\times10^{-1}$	1.57	$3.303\,9\times10^{-1}$	1.51
0.012 5	$6.857\,8\times10^{-1}$	2.38	$1.881\,2\times10^{-1}$	2.35	$6.566\,2\times10^{-2}$	2.33
0.006 25	$1.470\,1\times10^{-1}$	2.22	$4.055\,8\times10^{-2}$	2.21	$1.424\,3\times10^{-2}$	2.20
0.003 125	$3.489\,0\times10^{-2}$	2.08	$9.657\,8\times10^{-3}$	2.07	$3.403\,7\times10^{-3}$	2.07

**Table 3:** Convergence study for the advection equation for order 4 at $T=10$

*h*	$L^1$-error	Slope	$L^2$-error	Slope	$L^{\infty }$-error	Slope
0.05	$4.760\,1\times10^{0}$	–	$1.291\,9\times10^{0}$	–	$4.170\,2\times10^{-1}$	–
0.025	$3.167\,8\times10^{-1}$	3.91	$8.548\,2\times10^{-2}$	3.92	$2.921\,2\times10^{-2}$	3.84
0.012 5	$1.869\,8\times10^{-2}$	4.08	$5.123\,2\times10^{-3}$	4.06	$1.785\,0\times10^{-3}$	4.03
0.006 25	$1.142\,7\times10^{-3}$	4.03	$3.152\,7\times10^{-4}$	4.02	$1.107\,2\times10^{-4}$	4.01
0.003 125	$7.080\,4\times10^{-5}$	4.01	$1.959\,9\times10^{-5}$	4.01	$6.907\,0\times10^{-6}$	4.00

### Euler Equations

In this section, we first test our scheme on the following Euler equations in two dimensions:

25 $$\begin{aligned} \frac{\partial \textbf{u}}{\partial t}+ \dfrac{\partial \textbf{A}_1(\textbf{u})}{\partial x}+ \dfrac{\partial \textbf{A}_2(\textbf{u})}{\partial y} =0, \end{aligned}$$

where

$$\begin{aligned} \textbf{u}=(\rho , \rho \textbf{v}, E) \text { and }\textbf{A}=(\rho \textbf{v}, \rho \textbf{v}\otimes \textbf{v}+p\text {Id }, (E+p)\textbf{v})=(\textbf{A}_1,\textbf{A}_2). \end{aligned}$$

We run some standard cases: the isentropic case, the Sod problem, and a strong shock tube.

#### Isentropic Vortex

The first considered test case is the isentropic case. The boundary conditions are periodic. The initial conditions are given by

$$\begin{aligned} \rho&= \left[ 1 - \frac{(\gamma -1)\beta ^2}{32\gamma \uppi ^2}\exp \big (1 - r^2\big ) \right] ^{\frac{1}{\gamma -1}}, \\ v_x&= 1 - \frac{\beta }{4\uppi }\exp \left( \frac{1-r^2}{2}\right) (y-y_c), \\ v_y&= \frac{\sqrt{2}}{2}+ \frac{\beta }{4\uppi }\exp \left( \frac{1-r^2}{2}\right) (x-x_c), \\ p&= \rho ^\gamma , \end{aligned}$$

where $\gamma = 1.4$, $\beta =5,$ and $r=\sqrt{(x-x_c)^2+(y-y_c)^2}$. The computational domain is a square $[-10, 10]\times [-10,10]$. Also, the free stream conditions are given by

$$\begin{aligned} \rho _{\infty }=1,\quad v_{x,\infty }=1,\quad v_{y,\infty }=\frac{\sqrt{3}}{2},\quad p_{\infty }=1. \end{aligned}$$

The *y*-velocity is chosen such that the particle trajectories never coincide to a mesh point, if one start from a grid point initially. The final time is first set to $T=5$ for a convergence study (because of the cost mostly). The center of the vortex is set in $(x_c,y_c)=(Tv_{x,\infty },Tv_{y,\infty })$, modulo 20.

**Fig. 3:**
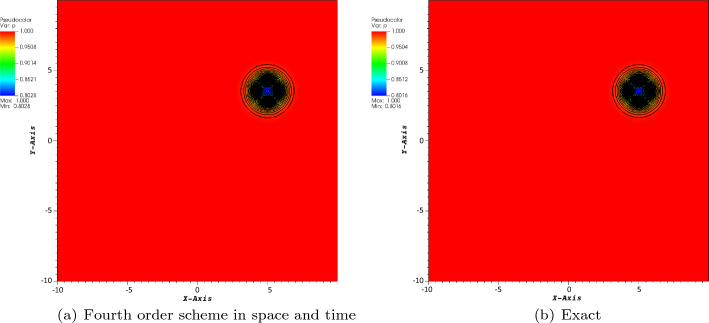
Plot of the pressure for the vortex problem at $T=5$

**Fig. 4:**
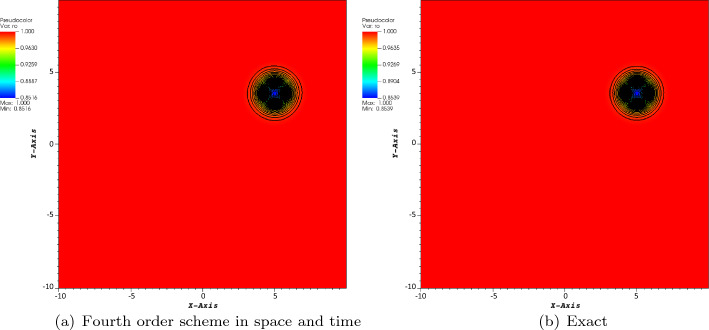
Plot of the density for the vortex problem at $T=5$

**Fig. 5:**
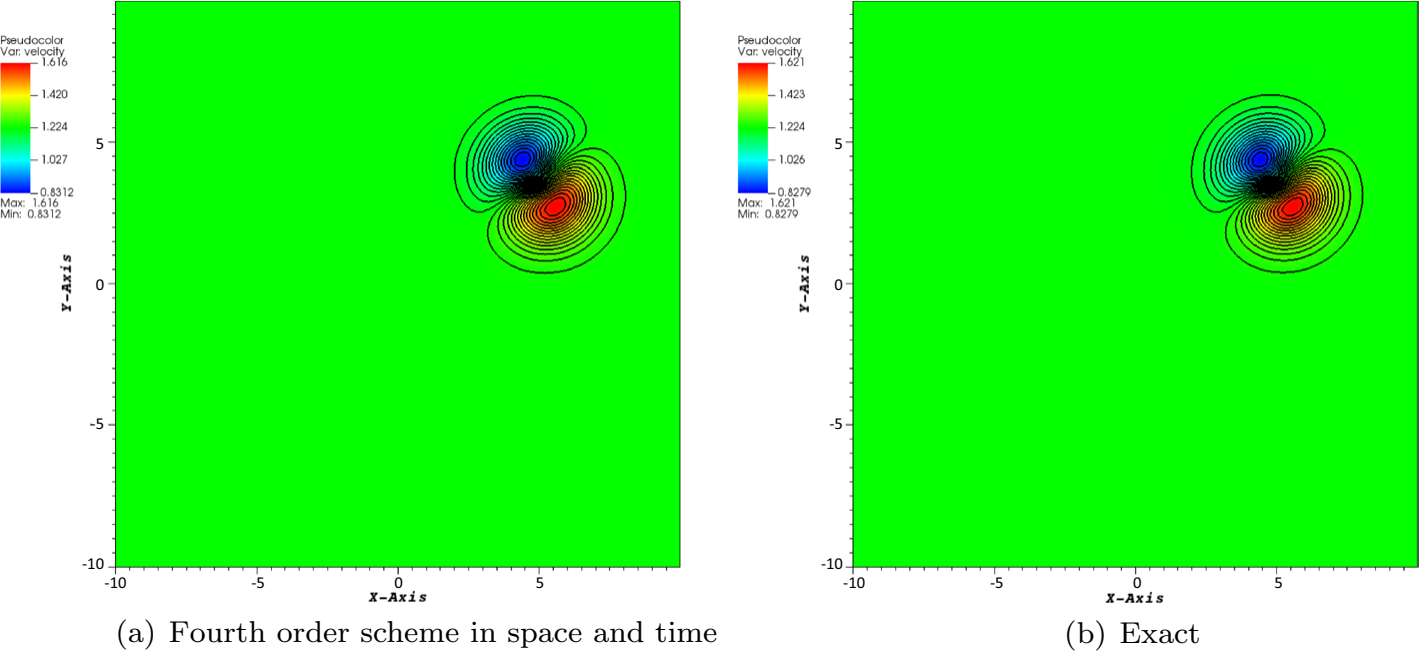
Plot of the norm of the velocity for the vortex problem at $T=5$

**Fig. 6:**
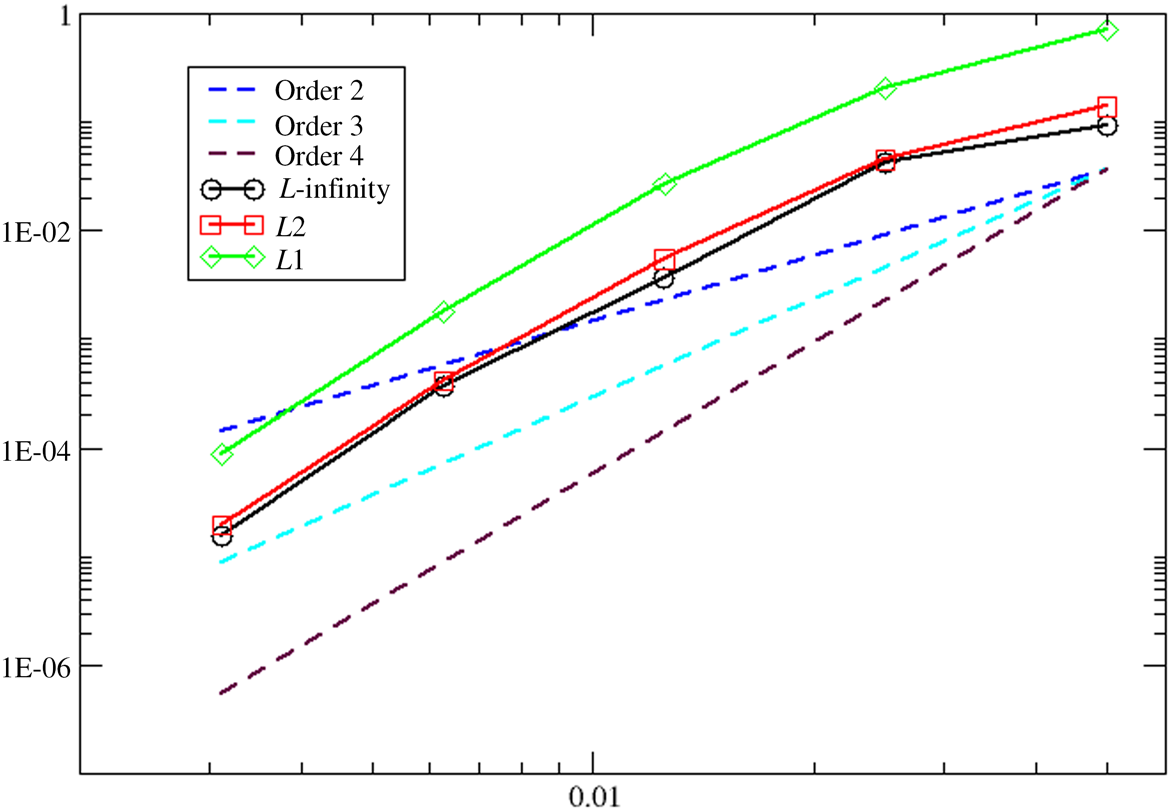
Convergence plot of density for the fourth order scheme in space and time at $T=5$

The reference solution is obtained on a regular Cartesian mesh consisting of $100\times 100$ elements and fourth-order scheme in space and time. The CFL number is set to 1, and we consider the four-wave model. In Figs. 3, 4, and 5, we have displayed the pressure, density, and norm of the velocity at $T=5$, respectively. We can observe that the plots show a very good behavior of the numerical scheme. The obtained convergence curves for the three considered error norms are displayed in Fig. 6 and show an excellent correspondence to the theoretically predicted order.

**Fig. 7:**
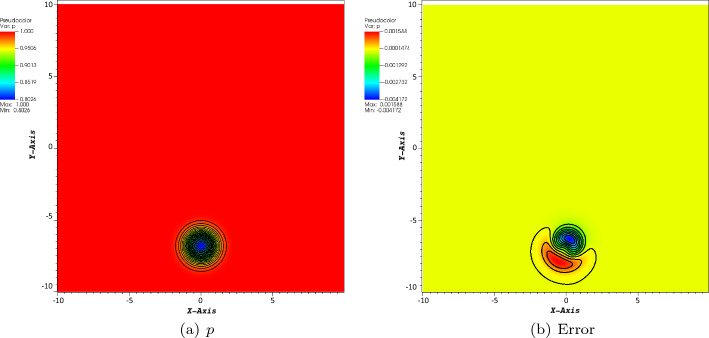
Pressure and error between the computed solution and the exact one at $T=200$ on a $200\times 200$ grid. We have $p_{i,j}-p^{ex}_{i,j}\in [-4.2\times 10^{-3}, 1.6\times 10^{-3}]$

In order to illustrate the long time behavior of the scheme, we show the pressure for $T=200$ and the error between the computed pressure and the exact one in Fig. 7 and a $200\times 200$ grid. Note that the typical time for a vortex to travel across the domain is about 10.

##### Remark 1

(About the stability condition) In this paper, we have focussed our attention on simulations with CFL=1. However, the stability analysis suggests that higher CFL can be used. In the case of the vortex case, using five iterations, we have been able to run this case, up to $T=200$ with CFL=1.2. This is smaller than what is suggested by Table 1. In this table, only the convection operator is considered, and we are not able to make an analysis where the source term is also included. It seems that the constraints are more severe than those suggested by the linear stability analysis.

#### Sod Test Case

**Fig. 8:**
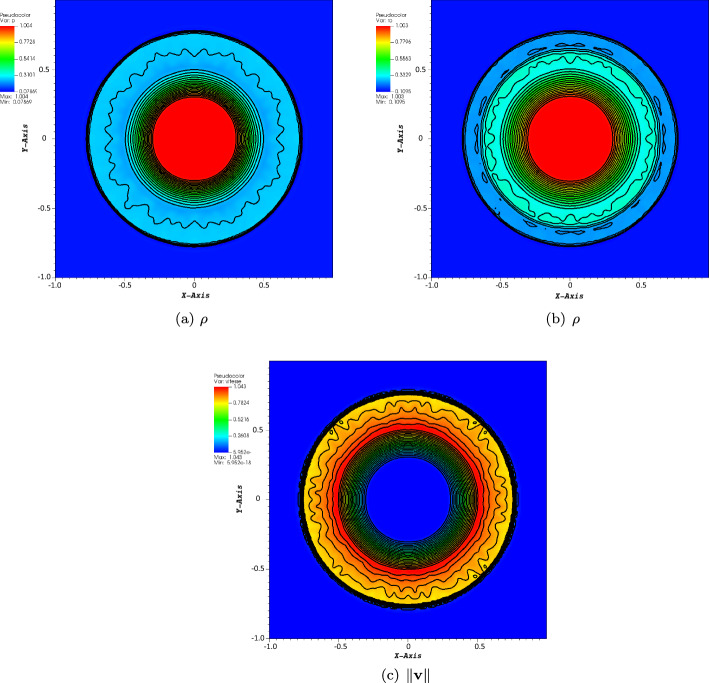
Sod problem, $T=0.16$ on a $200\times 200$ mesh with the limitation method

**Fig. 9:**
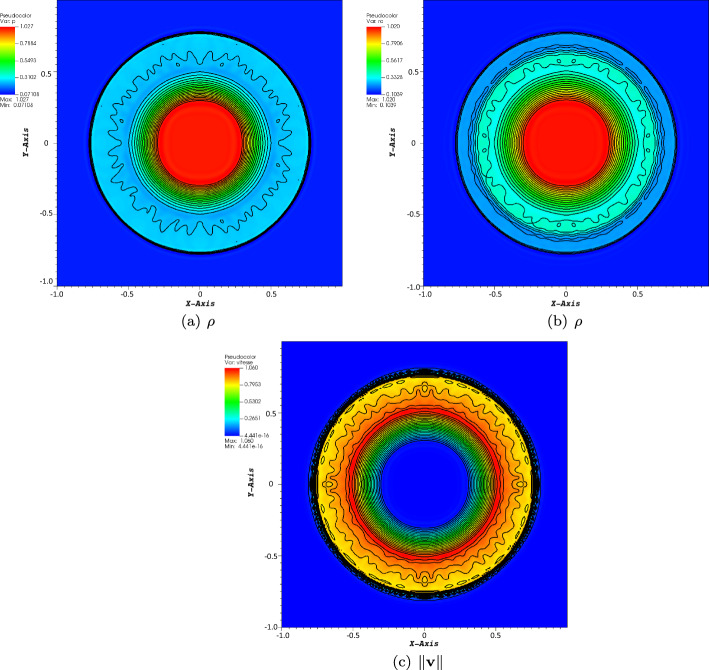
Sod problem, $T=0.16$ on a $200\times 200$ mesh with the MOOD method

Further, we have tested our high order kinetic scheme on a well-known two-dimensional Sod benchmark problem. This test is again solving the Euler equation (25). The domain is a square $[-1,1]\times [-1,1]$. The initial conditions are given by

$$\begin{aligned} (\rho _0,v_{x,0},v_{y,0},p_0) = {\left\{ \begin{array}{ll} ( 1, 0, 0, 1 ),&{} \text {if } r \leqslant 0. 5, \\ ( 0. 125, 0, 0, 0. 1 ),&{} \text {otherwise,} \end{array}\right. } \end{aligned}$$

and the boundary conditions are periodic. The final time is $T=0.16$ and the CFL number is set to 1. The two stabilisation methods have been tested and compared. The results for the limitation method are in Fig. 8, while the ones obtained with the MOOD method are displayed in Fig. 9. The two methods provide almost identical results. However, the MOOD method, for this case, never activates the first-order scheme, hence the results are obtained with the fourth order scheme. One can observe overshoots and undershoots at the shock, not strong enough to activate the first-order scheme. This drawback could be cured if one activates, in the MOOD method, the extrema detection procedure of [14] or [25]. When the limitation method is used, one can observe that the overshoot does not exist any more, while the undershoot is less important but still existing.

#### Strong Shock

The problem is defined on $[-1.5,1.5]\times [-1.5,1.5]$ for $T=0.025$. We had to use the MOOD technique to get the results, and the shocks are too strong.

26 $$\begin{aligned} (\rho _0,v_{x,0},v_{y,0},p_0)=\left\{ \begin{array}{ll} (1,0,0,1\,000)&{} \text { if } r \leqslant 0.5,\\ (1,0,0,1)&{} \text {else.} \end{array} \right. \end{aligned}$$

**Fig. 10:**
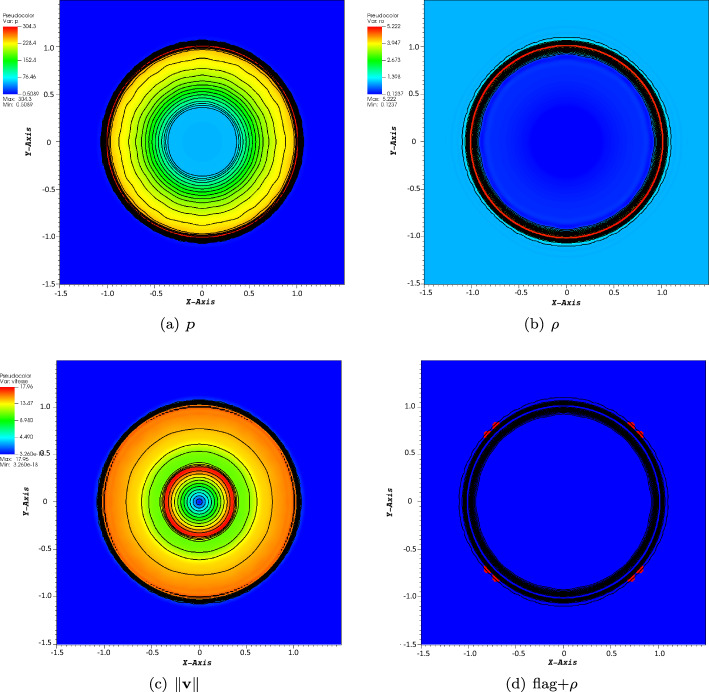
Result of case (26) on a $200\times 200$ grid, $\text{CFL}=1$, space order: 4, time order: 4, MOOD, final time

The pressure, density, and norm of the velocity are displayed in Fig. 10, for the final time. The simulation is done with CFL $=1$ on a $200\times 200$ grid. In Fig. 10(d), we show the iso-lines of the density (mostly to localize the strong features of the solution) and the elements where the formal accuracy is dropped to first order. These flagged elements are moving in time, and are always localized around the discontinuities of the solution. In most cases, only a very few elements are flagged.

## Conclusion

The purpose of this work is primarily to extend a class of kinetic numerical methods that can run at least at CFL one to the two-dimensional case. These methods can handle in a simple manner hyperbolic problems, and in particular compressible fluid mechanics. Our methodology can be arbitrarily high order and can use CFL numbers larger or equal to unity on regular Cartesian meshes. We have chosen the minimum number of waves, there are probably better solutions, and this will be the topic of further studies. These methods are not designed only for fluid mechanics, and other types of systems will be explored in the future. One interesting feature of these methods, working for CFL=1, is that the algebra for the streaming part of the algorithm can be made very efficient. This is an interesting feature.

## Footnotes

1 *x* is not a number if $x\ne x$.
